# Transglutaminase 2 exacerbates ovarian cancer survival by directly inactivating GSK3β

**DOI:** 10.1038/s41419-026-08447-0

**Published:** 2026-02-02

**Authors:** Ho Lee, Joon Hee Kang, Hyun Jung Kim, Kyun Heo, Mi Kyung Park, Jeong Hwan Park, Byung Il Lee, Jong In Yook, Soo-Youl Kim

**Affiliations:** 1 Division of Cancer Biology, National Cancer Center, Goyang, Gyeonggi Republic of Korea; 2 Graduate School of Cancer Science and Policy, National Cancer Center, Goyang, Gyeonggi Republic of Korea; 3 Department of Medicine, University of Ulsan College of Medicine, Seoul, Republic of Korea; 4 Biopharmaceutical Chemistry Major, School of Applied Chemistry, Kookmin University, Seoul, Republic of Korea; 5 Department of Biomedical Science, Hwasung Medi-Science University, Gyeonggi, Republic of Korea; 6 Division of Technology Convergence, National Cancer Center, Goyang, Gyeonggi Republic of Korea; 7 Department of Oral Pathology, Oral Cancer Research Institute, College of Dentistry, Yonsei University, Seoul, Republic of Korea

**Keywords:** Metastasis, Metastasis

## Abstract

Elevated expression of transglutaminase 2 (TGase 2, EC 2.3.2.13, protein-glutamine γ-glutamyltransferase, gene name *TGM2*) is known as one of the most upregulated genes during epithelial-mesenchymal transition (EMT) in ovarian cancer. Despite initial complete responses to conventional chemotherapy, ovarian cancer often recurs with metastasis, presenting a significant clinical challenge. Drug-resistant ovarian cancer cells exhibit markedly higher levels of TGase 2 compared to normal ovarian epithelium, which is associated with EMT activation, enabling them to evade chemotherapy effects. Intracellular TGase 2 is recognized as a key factor in maintaining the mesenchymal phenotype. Therefore, while EMT expression can be effectively reversed by inhibiting TGase 2, the underlying mechanism of this effect remains unclear. We found that TGase 2 promotes EMT by directly binding to glycogen synthase kinase-3β (GSK3β), promoting the stabilization of β-catenin. Domain mapping revealed that the N-terminus of TGase 2 interacts with the mid-region of GSK3β, leading to the autophagic degradation of GSK3β. Pharmacological disruption of this N-terminal interaction by streptonigrin, in combination with standard chemotherapy, extended overall survival in a xenograft model of ovarian cancer. This study identified TGase 2 as a pivotal regulator of EMT-driven metastasis and drug resistance.

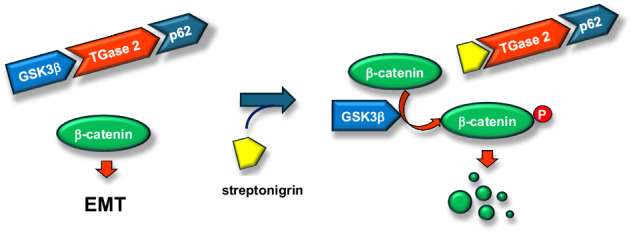

## Introduction

Ovarian cancer is one of the most commonly diagnosed cancers among women worldwide, with most patients dying from the disease within five years of diagnosis [1]. According to the 2020 World Health Organization (WHO) classification, epithelial ovarian cancer accounts for 90% of all cases. About 75% of epithelial ovarian cancer cases have frequent BRCA and p53 mutations, show rapid tumor growth, and are often detected in advanced stages (III or IV) [2]. Despite initial complete response rates exceeding 80% after frontline treatment, nearly 80% of patients with residual disease (>1 cm) relapse within 18 months, mainly due to chemoresistance [2–4]. These poor survival statistics are primarily caused by drug resistance and the activation of epithelial-mesenchymal transition (EMT) [5]. Current guidelines recommend platinum-based doublets, usually combined with paclitaxel or gemcitabine, followed by bevacizumab or poly(ADP-ribose) polymerase (PARP) inhibitors. However, these regimens rarely prevent recurrence or metastasis [6, 7]. Therefore, novel therapeutic strategies that can overcome chemoresistance and metastatic spread in ovarian cancer are urgently needed.

Transcriptomic comparisons of normal ovarian cells with ovarian cancer cells have identified transglutaminase 2 (TGase 2, EC 2.3.2.13; also known as transglutaminase 2, TGase C; gene name *TGM2*) [8] as one of the four upregulated genes in ovarian cancer, along with Wnt5a, OSF2, and PAI2 [9]. Immunohistochemistry showed positive TGase 2 staining in approximately 58% of ovarian cancer tissues, while expression in normal tissues was mostly negative [10]. Over the past few decades, TGase 2 has been extensively studied as a regulator of epithelial-mesenchymal transition (EMT). However, most research has focused on its extracellular (outside-in) signaling through interactions with fibronectin [11], integrins [12], or Wnt ligands at the cell surface [13]. Nevertheless, TGase 2 is also abundantly expressed in the cytoplasm and nucleus [8], suggesting that intracellular TGase 2 activity may play a more direct role in EMT and metastasis in ovarian cancer.

EMT-driven invasion involves three main stages: migration, adhesion, and proliferation. TGase 2 has been linked to each of these stages [8]. Although its role in cancer cell migration and invasion remains debated, *TGM2* knockdown (*TGM2* k/d) reduces the migration phenotype of breast cancer cells [9]. During adhesion, TGase 2 catalytic activity is not needed for cell adhesion to the extracellular matrix but is crucial for maintaining its association with assembled fibronectin fibers [10]. Key N-terminal residues (K30, R116, H134) mediate this interaction with the 45-kDa gelatin-binding domain of fibronectin (14,15) [11]. Small molecules that disrupt the TGase 2-fibronectin complex inhibit adhesion and metastasis [12]. In metastatic breast cancer cells, TGase 2 interacts with b1, b4, and b5 integrins, thereby facilitating extracellular matrix (ECM) binding, promoting EMT, and activating outside-in signaling [13, 14]. TGase 2 also directly activates β-catenin signaling by binding to LRP5/6 (low-density lipoprotein receptor-related proteins 5 and 6) [15], highlighting its extracellular role in the EMT process. During the proliferation stage, TGase 2 activates TGF-β (transforming growth factor-β) in the ECM [16] and maintains persistent NF-κB activation intracellularly [17]. Many cytotoxic agents inadvertently activate NF-κB and its pro-survival targets, contributing to chemoresistance [18]. TGase 2 promtes this signaling by cross-linking I-κBα and targeting it for depletion [19], and the specific cross-linking sites on I-κBα were confirmed by site-directed mutagenesis [20]. In ovarian cancer cells, TGase 2 overexpression reversed cisplatin-induced cell death via NF-κB activation [21], whereas TGase 2 knockdown markedly enhanced the efficacy of paclitaxel in chemoresistant cells [22]. Collectively, these findings establish TGase 2 as a key regulator of both EMT and drug resistance in ovarian cancer. The current study explores two important questions. First, since the RTK-GSK3β-β-catenin signaling pathway is well known as a primary EMT regulatory axis, how can the role of TGase 2 in EMT be justified? Second, what is the mechanism by which TGase 2 promotes EMT at the cellular level? Since most TGase 2 is localized to the cytoplasm [23]. Answering these questions will clarify the multifaceted role of TGase 2 and help identify new therapeutic opportunities to overcome chemotherapy resistance and metastasis in ovarian cancer.

## Results

### TGase 2 expression correlates with ovarian cancer progression and metastasis

**Fig. 1:**
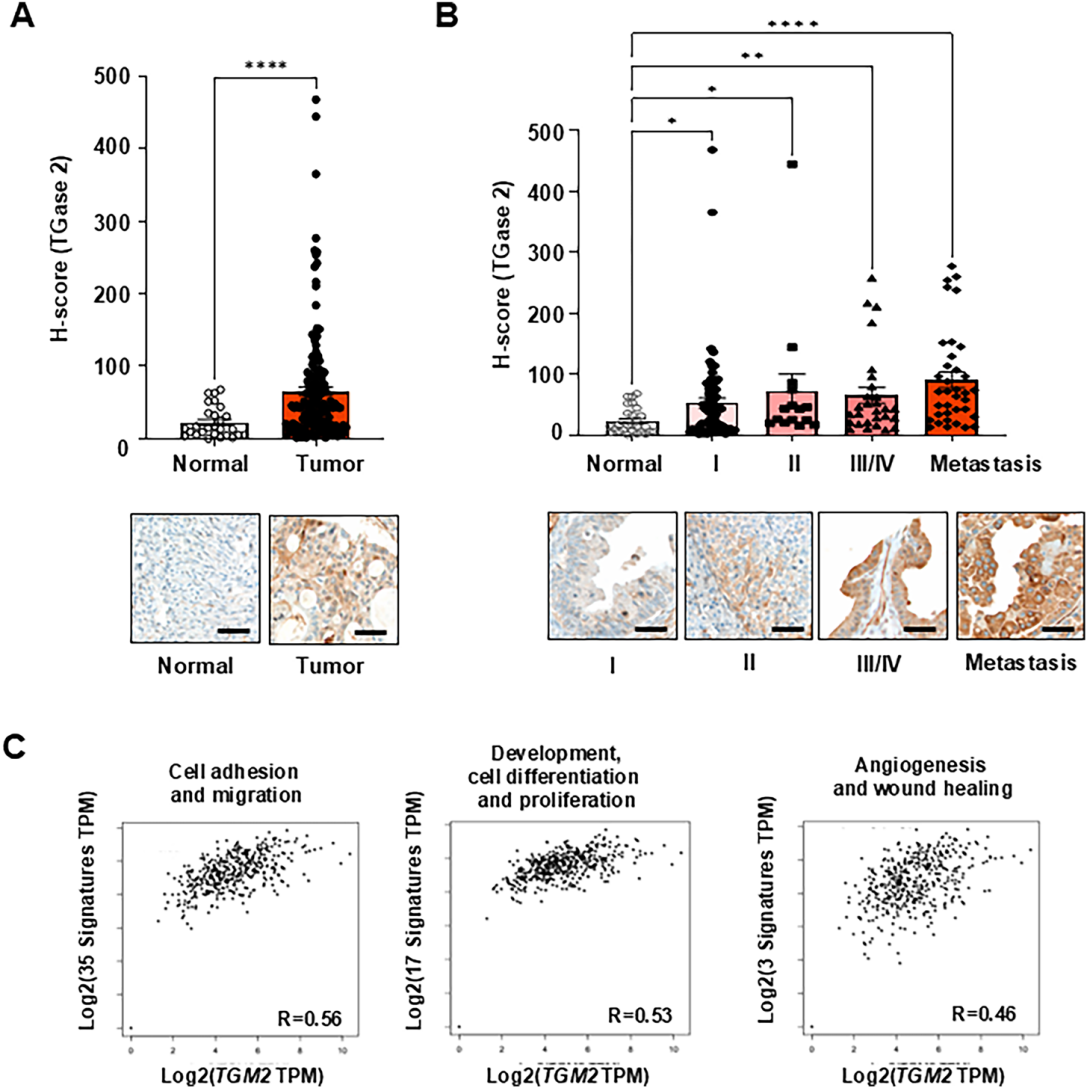
TGase 2 is upregulated in metastatic ovarian cancer. **A** Quantification of histological (H) scores for transglutaminase 2 (TGase 2) in normal ovarian tissue (*n* = 27) and primary ovarian tumors (*n* = 180) using inForm™ image‑analysis software. Scale bar, 100 µm. **B** Quantitative data for TGase 2 staining on tissue microarrays from normal controls (*n* = 27) and tumors classified as stage I (*n* = 83), stage II (*n* = 24), stage III–IV (*n* = 37), or metastatic lesions (*n* = 36). Representative immunohistochemical (IHC) images are shown Supplementary Fig. S1A. Scale bar, 100 µm. **C** Correlation analysis between *TGM2* and epithelial-to-mesenchymal transition (EMT)–related genes grouped by biological function. The graphs of TMA analysis are presented as mean ± standard error of the mean (SEM). More information on this cohort is provided in Supplementary Fig. 1A. Comparisons between two groups were performed using the Mann–Whitney test, while one-way analysis of variance (ANOVA) was used for comparisons among three or more groups. Statistical significance was defined as *p < 0.05, **p < 0.01, ****p < 0.0001.

To assess the relationship between TGase 2 expression and ovarian cancer development and metastasis, we conducted immunohistochemistry on a high-density ovarian cancer tissue array (OV208b, US Biomax). This array includes 207 cores representing major histopathological subtypes: mucinous adenocarcinoma, mucinous papillary adenocarcinoma, serous adenocarcinoma, serous papillary adenocarcinoma, and metastatic carcinoma, along with 27 morphologically normal ovary samples adjacent to tumor tissue. The staged distribution of cancer tissues across these subtypes allowed for a systematic evaluation of TGase 2 expression relative to ovarian cancer progression and malignancy. The average immunohistochemical H-score for TGase 2 in normal ovarian tissue was 22.6, which increased to 65.3 in ovarian cancer samples, representing approximately a 2.9-fold rise (Fig. 1A and Supplementary Fig. 1A). When grouped by clinical stage, TGase 2 H-scores were 52.2 for stage I, 71.5 for stage II, 65.0 for stages III–IV combined, and 90.1 for metastatic lesions, indicating a gradual increase in expression with disease progression (Fig. 1B). To explore the link between *TGM2* and EMT-related genes, we classified EMT-associated genes into three functional categories: (1) cell adhesion and migration, (2) development/cell differentiation and proliferation, and (3) angiogenesis and wound healing (Fig. 1C). The classification was based on the study by Gröger et al. [24]. Correlation analysis revealed strong positive correlations between *TGM2* expression and EMT-related genes, with Pearson coefficients of 0.56 for cell adhesion and migration, 0.53 for development/cell differentiation and proliferation, and 0.46 for angiogenesis and wound healing (Fig. 1C and Supplementary Fig. 1B). These findings suggest that *TGM2* expression is closely linked to a wide range of EMT-associated genes, supporting its central role in EMT regulation.

### TGase 2 Binds to GSK3β and regulates its stability via autophagic degradation

**Fig. 2:**
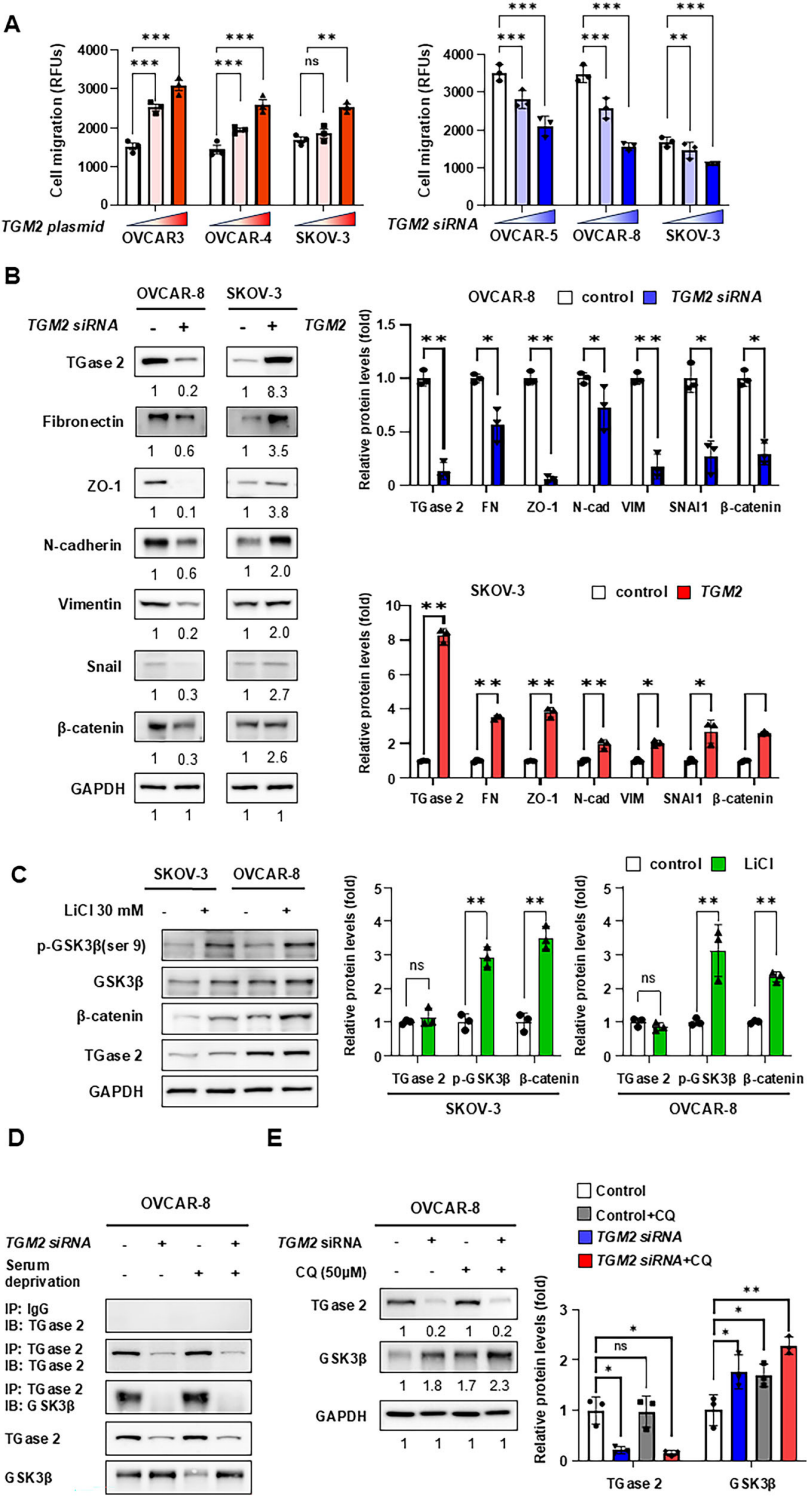
TGase 2 interacts with GSK3β and regulates its stability. **A** TGase 2 modulates ovarian cancer cell migration. OVCAR‑5, OVCAR‑8, and SKOV‑3 cells were transfected for 48 h with *TGM2* siRNA (20 or 40 nM), after which their migratory capacity was measured. Simultaneously, OVCAR‑3, OVCAR‑4, and SKOV‑3 cells were transfected with *TGM2* expression plasmids (2 or 4 µg) for 48 h and tested in the same assay. RFUs indicate relative fluorescence units. **B** Effect of TGase 2 on epithelial-to-mesenchymal transition (EMT) markers. OVCAR‑8 cells transfected with *TGM2* siRNA (40 nM) and SKOV‑3 cells transfected with *TGM2* plasmid (4 µg, each for 48 h) were analyzed by immunoblotting for EMT-associated proteins. **C** WNT/β‑catenin signaling in ovarian cancer cells. OVCAR‑8 and SKOV‑3 cells were treated with lithium chloride (LiCl; 30 mM, 8 h) to inhibit GSK3β and activate the β‑catenin pathway. **D** TGase 2–GSK3β interaction detected by co‑immunoprecipitation. OVCAR‑8 cells transfected with *TGM2* siRNA (40 nM) for 48 h were lysed under serum‑supplemented or serum‑starved conditions before co‑immunoprecipitation. **E** TGase 2 regulates GSK3β turnover through autophagy. OVCAR‑8 cells transfected with *TGM2* siRNA (40 nM) for 48 h were starved and then treated with chloroquine (CQ; 50 µM, 6 h) or left untreated, followed by immunoblot analysis. All graphs are displayed as mean ± standard deviation (SD). All experiments were performed with three samples per group (*n* = 3). Comparisons between two groups were performed using the Student’s *t* test, whereas comparisons among three or more groups were conducted using one-way analysis of variance (ANOVA). Statistical significance was defined as *p < 0.05 or **p < 0.01 or ***p < 0.001, and ns indicates not significant.

Our findings align with previous reports, which show that TGase 2 expression is elevated in metastatic ovarian cancer tissues [22, 25–27]. To assess the effect of TGase 2 on cell migration, we performed a dose-dependent siRNA knockdown of *TGM2* in the TGase 2-high ovarian cancer cell lines OVCAR-5 and OVCAR-8, and a dose-dependent overexpression in the TGase 2-low lines OVCAR-3 and OVCAR-4. We also used both gain and loss-of-function approaches on SKOV-3 cells (Fig. 2A). The impact of TGase 2 expression on cell migration was measured with a fibronectin-coated cell migration assay. Higher TGase 2 levels increased ovarian cancer cell migration, while reducing TGase 2 decreased migration. Specifically, migration through fibronectin-coated membranes increased by 1.7- to 2.0-fold in OVCAR-3, 1.5- to 1.9-fold in OVCAR-4, and 1.1- to 1.5-fold in SKOV-3 upon TGase 2 overexpression. Conversely, *TGM2* knockdown lowered migration by 21–40%, 29–54%, and 8–32% in OVCAR-5, OVCAR-8, and SKOV-3, respectively. These results suggest a link between TGase 2 levels and cell migration, an essential first step in metastasis.

Next, we examined whether TGase 2 expression affects the regulation of EMT markers during ovarian cancer cell migration. In OVCAR-8 cells, knocking down *TGM2* resulted in decreased levels of Fibronectin, ZO-1, N-cadherin, Vimentin, Snail, and β-catenin. Conversely, overexpressing *TGM2* in SKOV-3 cells caused an increase in all these EMT markers (Fig. 2B). To determine if this effect involves WNT/β‑catenin signaling, cells were treated with lithium chloride (LiCl, 30 mM, 8 h), a GSK3β inhibitor that stabilizes β‑catenin. LiCl increased β‑catenin levels without affecting TGase 2 levels (Fig. 2C). However, *TGM2* knockdown reduced the induction of EMT markers, indicating that TGase 2 impacts the WNT/β‑catenin pathway in ovarian cancer (Fig. 2B and C). Co-immunoprecipitation experiments demonstrated that TGase 2 directly interacts with GSK3β, and reducing TGase 2 expression resulted in a decrease in the formation of the TGase 2-GSK3β complex (Fig. 2D). Small interfering RNA–mediated *TGM2* knockdown markedly decreased the formation of the TGase 2–GSK3β complex. We previously demonstrated that TGase 2 induces autophagic degradation of the tumor suppressor p53 [28]. To investigate whether TGase 2 similarly regulates GSK3β through autophagic degradation, OVCAR-8 cells were treated with chloroquine, a lysosomotropic autophagy inhibitor. Chloroquine alone increased GSK3β protein levels by 1.5-fold, while combining it with TGase 2 siRNA resulted in the most significant stabilization, raising GSK3β by approximately 2.3-fold compared to control (Fig. 2E). Overall, our results indicate that TGase 2 promotes EMT and migration in ovarian cancer by binding to and destabilizing GSK3β. As a result, loss of TGase 2 relieves GSK3β suppression, inhibits WNT/β‑catenin signaling, and may reverse metastatic progression.

### Deletion of the TGase 2 N-terminus restored GSK3β levels

**Fig. 3:**
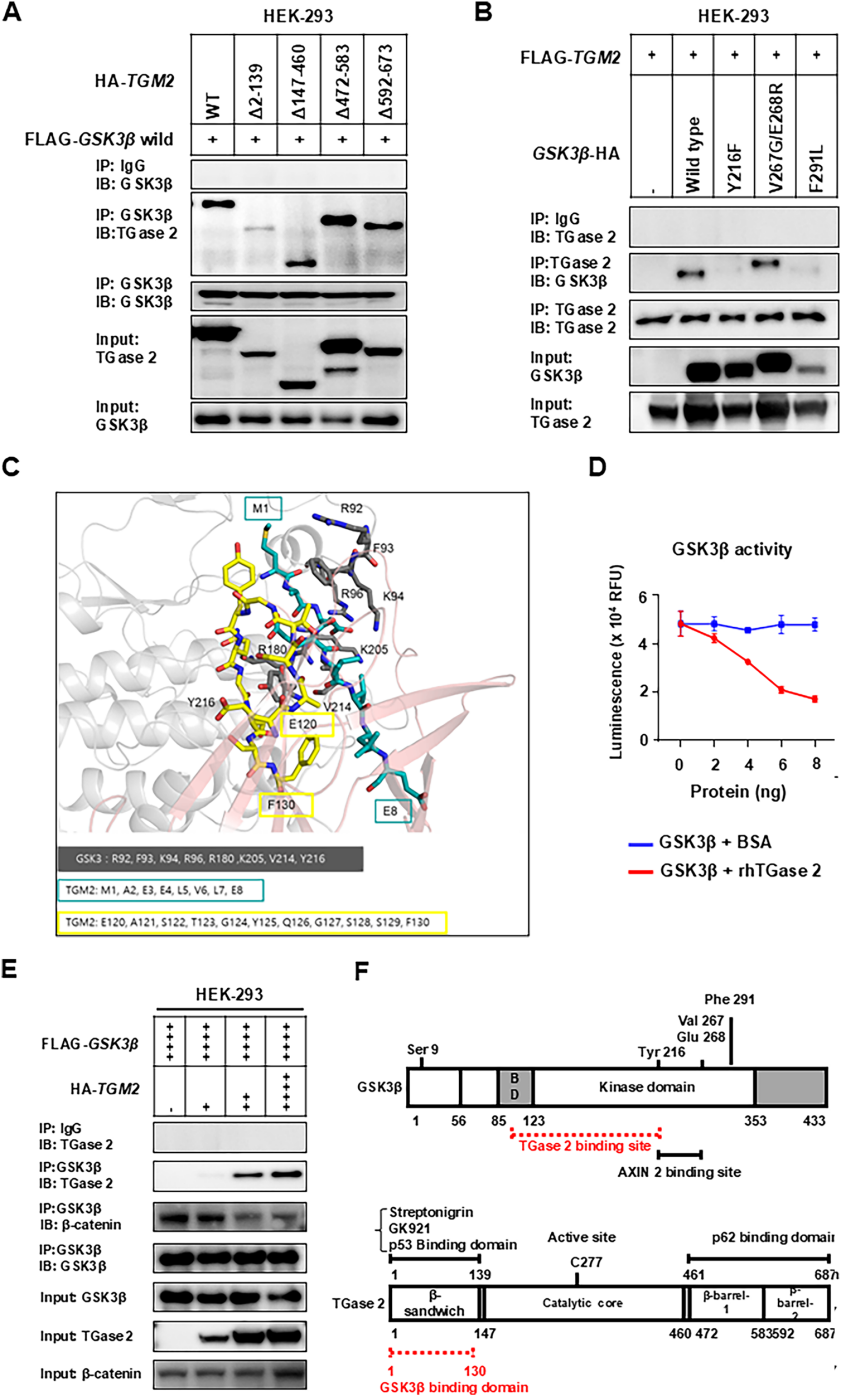
The N-terminus of TGase 2 is essential for binding GSK3β. **A** N-terminal region of TGase 2 is required for its interaction with GSK3β. HEK-293 cells were co-transfected with plasmids encoding FLAG-GSK3β and either wild-type TGM2 or the indicated TGM2 deletion mutants (Δ2-139, Δ147-460, Δ472-583, Δ592-673). Complexes were immunoprecipitated using an anti-FLAG antibody and analyzed by immunoblotting. **B** TGase 2 binds the catalytic core of GSK3β. HEK-293 cells were transfected with plasmids encoding FLAG-TGM2 and either wild-type GSK3β or point-mutated variants (Y216F, V267G/E268R, or F291L). Protein complexes were purified via anti-FLAG immunoprecipitation and examined by immunoblot analysis. **C** In silico docking predicts a direct interaction between TGase 2 and GSK3β. ClusPro docking of GSK3β (PDB 4NM5, gray) with TGase 2 (PDB 2Q3Z, pink) is shown as cartoons; interface residues are displayed as sticks (GSK3β, gray; TGase 2, blue-green and yellow). **D** TGase 2 inhibits the kinase activity of GSK3β in a dose-dependent manner. Recombinant GSK3β (2 ng) was incubated with increasing amounts of rhTGase 2 (0–8 ng), and kinase activity was quantified. The graphs display mean ± standard deviation (SD). Assays were performed with three samples per group (*n* = 3). rhTGase 2; recombinant human TGase 2. **E** The TGase 2:GSK3β ratio modulates competing protein-protein interactions. Elevated TGase 2 expression strengthens the TGase 2–GSK3β interaction while reducing the association between GSK3β and β‑catenin. **F** Domain organization of GSK3β and TGase 2. Schematic diagrams depict the primary structures and domains of each protein, with residue ranges corresponding to the constructs analyzed in this study. BD: substrate binding domain.

To identify the region of TGase 2 required for GSK3β binding, a series of HA-tagged TGase 2 deletion constructs was co-transfected with FLAG-GSK3β into HEK-293 cells (Fig. 3A). Immunoprecipitation with an anti-HA antibody showed that deleting the N-terminal 1–139 amino acids completely abolished TGase 2-GSK3β interaction, while deletions including residues 147-460, 472-583, or 592-673 had no effect. Therefore, the N-terminus of TGase 2 is essential for the interaction and for maintaining low cellular GSK3β levels. To further investigate whether TGase 2 contacts the catalytic core of GSK3β, three-point mutants within the kinase domain (Y216F, V267G/E268R, F291L) were examined (Fig. 3B). The Y216F and F291L mutants did not co-precipitate with TGase 2, whereas V267G/E268R bound normally, indicating that Tyr216 and Phe291 are key contact residues. This shows that specific residues within the GSK3β kinase domain are critical for interaction with TGase 2.

To obtain a more detailed view of the binding interface between TGase 2 and GSK3β, we used the ClusPro server (Fig. 3C and Supplementary Fig. 2A). ClusPro predicts complex structures by (1) sampling billions of conformations using a Fast Fourier Transform algorithm implemented in PIPER, (2) clustering the 1000 lowest-energy structures based on root-mean-square deviation (RMSD) values, and (3) refining selected complexes through energy minimization 25, 26. The predicted GSK3β-TGase 2 complex revealed extensive interactions between the GSK3β kinase domain and the β-sandwich domain of TGase 2 (Fig. 3C). Eight GSK3β residues (Arg92, Phe93, Lys94, Arg96, Arg180, Lys205, Val214, Tyr216) and 19 TGase 2 residues (Met1, Ala2, Glu3, Glu4, Leu5, Val6, Leu7, Glu8, Glu120, Ala121, Ser122, Thr123, Gly124, Tyr125, Gln126, Gly127, Ser128, Ser129, Phe130) were identified as critical for their interaction, consistent with the mutagenesis data (Supplementary Fig. 2B). Based on the finding that the N-terminal domain of TGase 2 interacts with the kinase domain of GSK3β, we next investigated whether increased TGase 2 expression affects GSK3β kinase activity using in vitro recombinant protein assays (Fig. 3D). While higher concentrations of BSA had no effect on GSK3β kinase activity, equimolar or higher amounts of TGase 2 gradually inhibited enzyme activity. To assess whether the interaction between TGase 2 and GSK3β depends on their relative levels, we co-transfected HEK-293 cells with HA-tagged TGase 2 and FLAG-tagged GSK3β at increasing TGase 2:GSK3β plasmid ratios. Higher TGase 2 expression proportionally increased its binding to GSK3β. Conversely, when the TGase 2:GSK3β ratio reached 1:2 and 1:1, the interaction with GSK3β decreased by 45% and 52%, respectively (Fig. 3E). These results suggest that elevated TGase 2 levels inhibit GSK3β‘s enzymatic activity, thereby promoting ovarian cancer metastasis. Overall, our immunoprecipitation results and structural modeling offer a detailed view of the TGase 2-GSK3β binding (Fig. 3F).

### TGase 2 loss extends survival and reduces metastasis in vivo

**Fig. 4:**
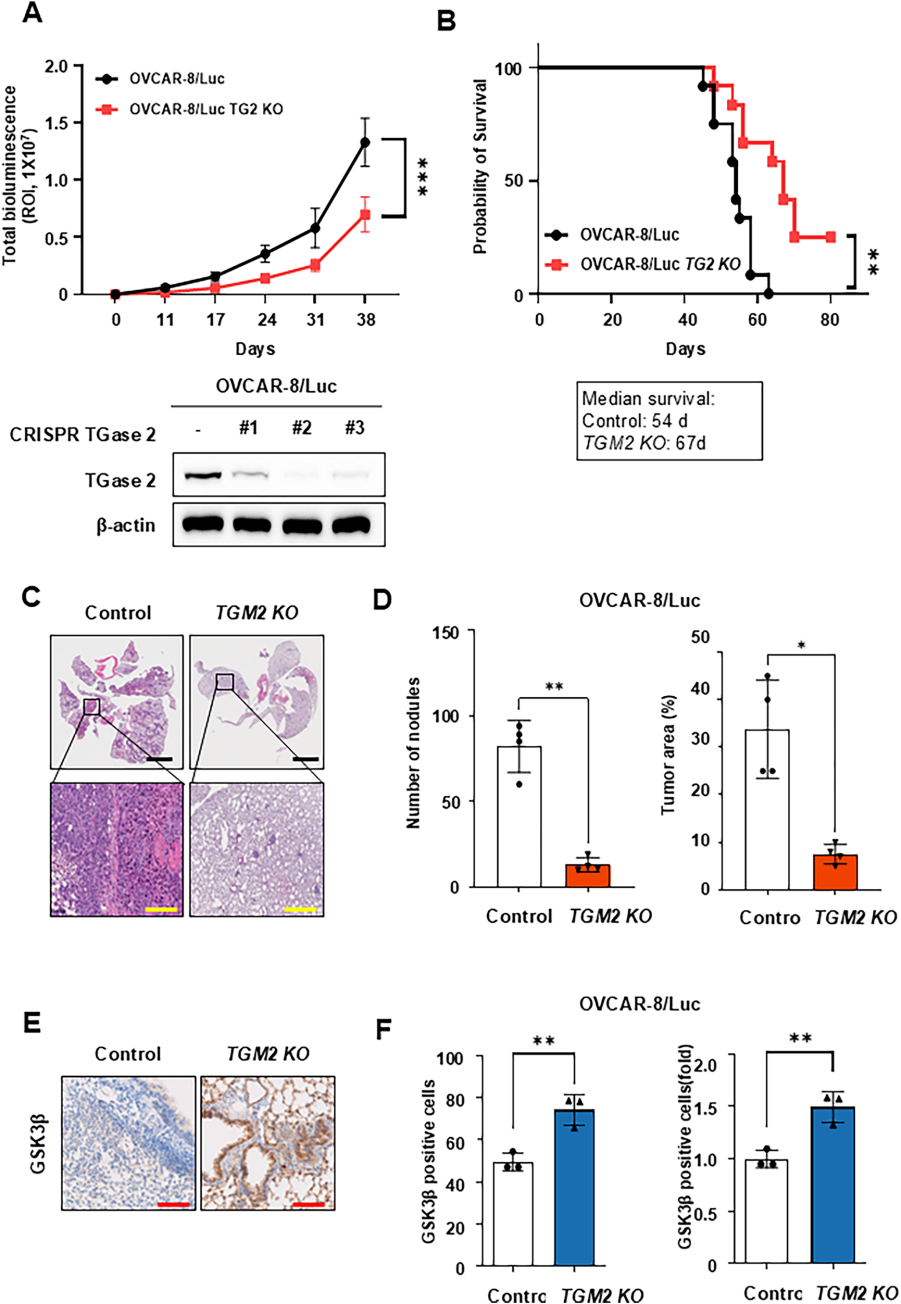
*TGM2* knockout (*TGM2* KO) reduces ovarian cancer progression and extends survival. **A** Generation of *TGM2* knock-out cell lines using the CRISPR system. TGase 2 expression was confirmed by immunoblotting, and clone #2, which showed the lowest TGase 2 level, was selected for in vivo studies. To mimic ovarian cancer growth and metastasis, we evaluated antitumor efficacy using an intraperitoneal orthotopic model. Loss of TGase 2 decreases intraperitoneal (i.p.) tumor growth in an OVCAR‑8/Luc xenograft model. BALB/c‑nu/nu mice (*n* = 12 per group) were inoculated i.p. (intraperitoneally) with 1 ×10^7^ parental OVCAR‑8/Luc cells or OVCAR‑8/Luc cells with *TGM2* KO. Tumor burden was monitored weekly via in vivo bioluminescence imaging (IVIS) and measured as total photon flux using Living Image software. Graphs show mean ± standard deviation (SD). Comparisons between groups used a Two-way ANOVA. Statistical significance was set at ***p < 0.001. ROI, region of interest. **B** Kaplan–Meier survival curves for the cohorts in (**A**). Deletion of TGM2 significantly improved overall survival (**p < 0.01, log‑rank test). To examine if TGase 2 also plays a role in late metastatic stages, we used a tail-vein lung metastasis model. TGase 2 promotes experimental ovarian cancer metastasis. Parental or *TGM2*‑KO OVCAR‑8/Luc cells were injected into the tail vein of BALB/c‑nude mice (*n* = 4 per group). **C** Metastatic lesions collected at the endpoint were analyzed through immunohistochemistry. Scale bar, 100 μm (yellow) and 2 mm (black). **D** Quantification of metastatic burden and nodules. *p < 0.05, **p < 0.01. **E**, **F** Immunohistochemical analysis of GSK3β expression in TG2-deficient tumors was performed using the Vectra Polaris™ Automated Quantitative Pathology Imaging System and inForm software (Akoya Biosciences, Waltham, MA, USA). Scale bar, 100 μm. Bar graphs display the number of metastatic nodules and the total tumor area per mouse (mean ± SD; *n* = 4). **p < 0.01.

To investigate the effect of TGase 2 in ovarian cancer progression in vivo, luciferase-labeled OVCAR‑8 cells (OVCAR‑8/Luc) or their *TGM2*‑knockout derivatives (*TGM2* KO) were injected intraperitoneally into BALB/c‑nude mice. Longitudinal bioluminescence imaging showed significantly reduced tumor growth in the *TGM2* KO group (Fig. 4A). Consistent with this slower tumor progression, median survival increased by 13 days in *TGM2* KO mice (Fig. 4B). In a separate group, mice received tail‑vein injections to simulate hematogenous spread. At the study’s end, hematoxylin‑and‑eosin lung sections showed markedly fewer and smaller metastatic foci in *TGM2* KO mice: pulmonary nodule count and total tumor area were reduced by 85% and 80%, respectively (Fig. 4C and D). To assess whether the decreased lung metastasis in *TGM2* KO mice correlated with increased GSK3β expression, immunohistochemical staining was performed. Results indicated GSK3β levels in lung metastatic lesions of *TGM2* KO mice were about 50% higher than in the control group (Fig. 4E and F). Overall, these in vivo results demonstrate that the loss of TGase 2 inhibits metastatic colonization and enhances survival, supporting the idea that TGase 2 acts as a critical negative regulator of GSK3β, with its loss reactivating anti-metastatic pathways.

### Pharmacological inhibition of TGase2 stabilizes GSK3β

**Fig. 5:**
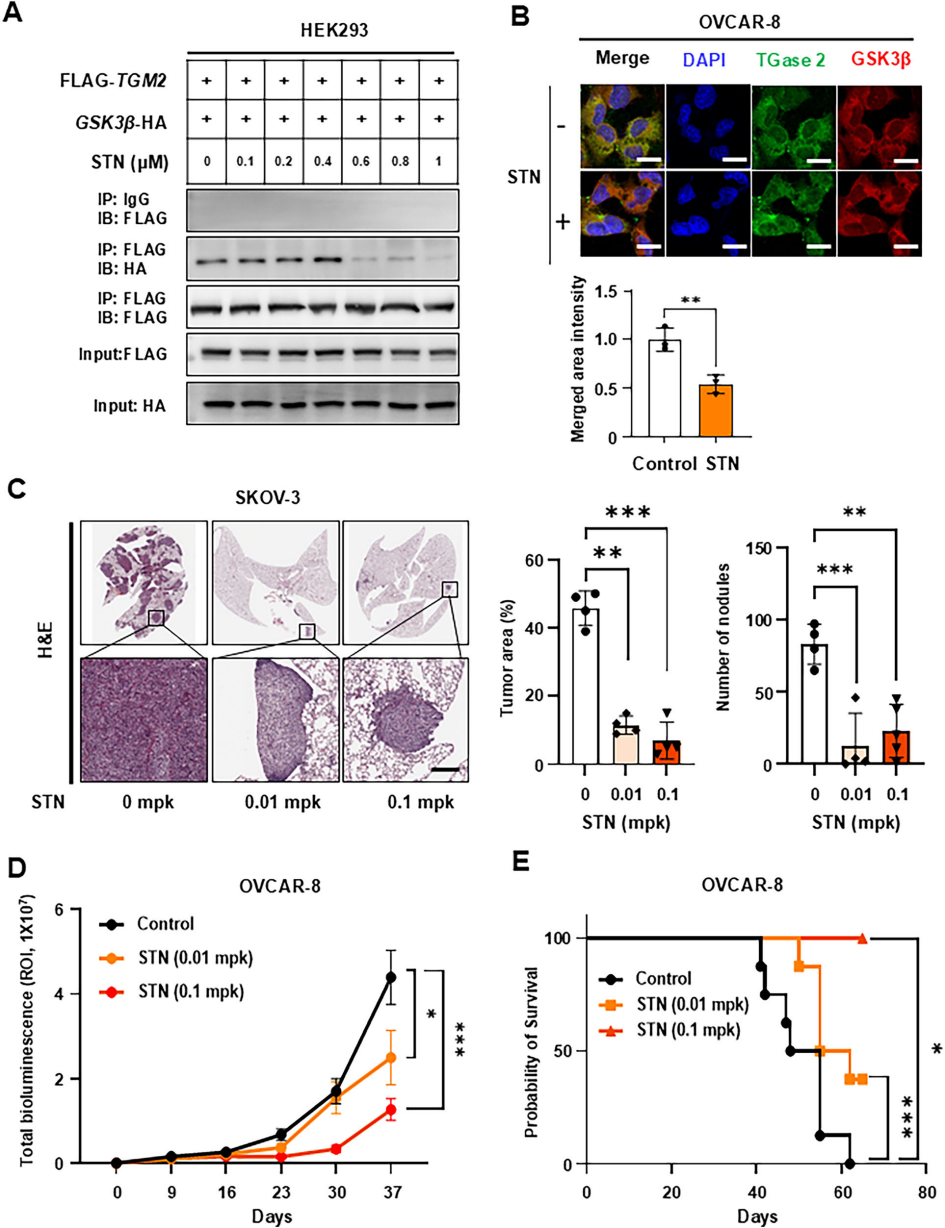
Pharmacological inhibition of TGase2 stabilizes GSK3β and suppresses ovarian cancer progression in vivo. **A** Streptonigrin (STN) disrupts the TGase2–GSK3β interaction. HEK-293 cells were co‑transfected with FLAG-*TGM2* and *GSK3β*-HA plasmids, cultured for 48 h, and then treated with the indicated concentrations of STN for 1 h. **B** STN reduces intracellular co‑localization of TGase 2 and GSK3β. OVCAR‑8 cells were exposed to 500 nM STN for 1 h and analyzed by confocal microscopy. Scale bar, 20 µm. **C** To assess whether TGase 2 inhibition suppresses metastasis, we utilized a tail-vein lung metastasis model. TGase2 inhibition decreases metastatic burden in a tail‑vein ovarian cancer model. SKOV‑3 cells were injected into the tail vein of BALB/c‑nude mice (*n* = 4 per group). Metastatic lesions were collected and analyzed via immunohistochemistry. Scale bar, 100 µm. Bar graphs represent the number of metastatic nodules and total tumor area (mean ± SD; *n* = 4). Scale bar, 200 µm. **D** To confirm whether the same effect is observed in an authentic ovarian cancer metastasis model, we revalidated it using an orthotopic model. TGase2 inhibition delays tumor growth in an intraperitoneal xenograft model. OVCAR‑8/Luc cells (1 × 10⁷) were injected intraperitoneally into mice (*n* = 9 per group). Beginning two days before cell inoculation, mice received oral PBS or STN (0.01 or 0.1 mg/kg) five days per week. Tumor growth was monitored weekly by bioluminescence imaging (IVIS) and total photon flux was plotted (mean ± SEM). **E** Kaplan–Meier survival curves for the mice in Fig. 4D over 80 days after cell injection. All graphs are presented as mean ± standard deviation (SD). Multiple comparisons among three or more groups were performed using one-way analysis of variance (ANOVA). Statistical significance was considered at *p < 0.05, **p < 0.01, and ***p < 0.001.

We previously reported that p53 binds to the N-terminus of TGase 2, directing it to autophagosomes and promoting tumor survival. Streptonigrin has been reported to target this N-terminal region and exhibits anticancer activity [28, 29]. To investigate whether streptonigrin disrupts the TGase 2-GSK3β interaction, co-immunoprecipitation assays were performed on HEK-293 cells co-transfected with TGase 2 and GSK3β after treatment. Streptonigrin concentrations as low as 0.6 mM robustly inhibited TGase 2-GSK3β binding (Fig. 5A). In experiments with recombinant proteins of TGase 2 and GSK3β, streptonigrin also effectively disrupted the TGase 2-GSK3β interaction (Supplementary Fig. 3A). Immunofluorescence analysis in OVCAR-8 cells revealed that treatment with 500 nM streptonigrin reduced the co-localization ratio of TGase 2 and GSK3β by 50% compared with the control group (Fig. 5B and Supplementary Fig. 3B). The effect of streptonigrin on the invasion of ovarian cancer cells, including SKOV-3 and OVCAR-8, was determined by the migration assay (Supplementary Fig. 3C and D). TGase 2 inhibitor suppressed migration and invasion of SKOV-3 and OVCAR-8 cells. Together with data from purified proteins, these findings indicate that small‑molecule blockade of the TGase 2 N‑terminus can release and stabilize GSK3β.

### TGase 2 inhibition potentiates standard chemotherapy

**Fig. 6:**
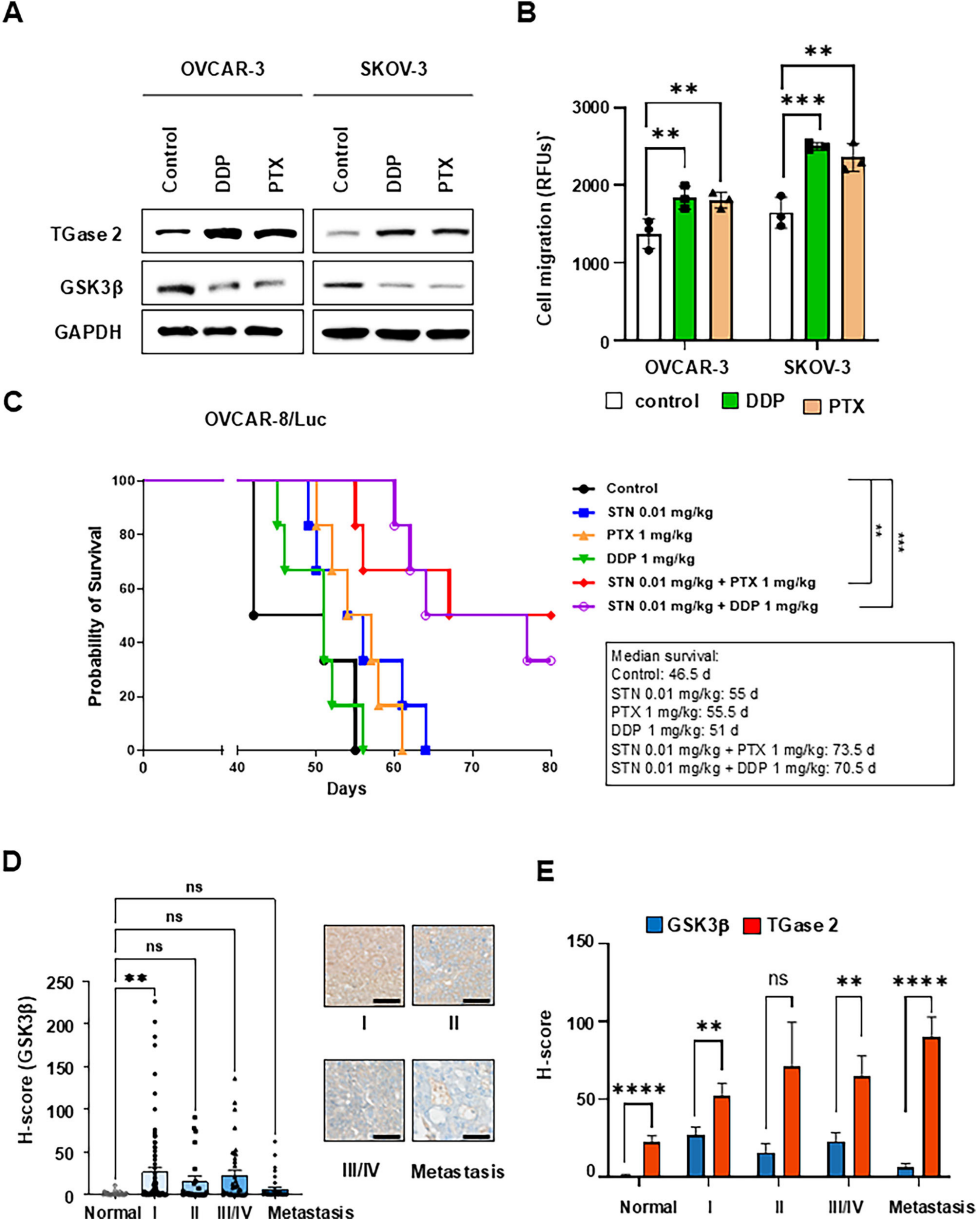
The combination of streptonigrin with conventional chemotherapies extends median survival in an ovarian cancer model. **A** Conventional chemotherapies increase TGase 2 levels and decrease GSK3β levels. OVCAR‑3 or SKOV‑3 cells were treated with cisplatin (DDP, 1 mM) or paclitaxel (PTX, 10 μM) for 24 h, after which TGase 2 and GSK3β protein levels were measured by immunoblotting. **B** Chemotherapy treatment enhances the migratory ability of ovarian cancer cells. Cell migration was quantified after treatment with DDP or PTX in OVCAR-3 and SKOV-3 cells. The graphs show mean ± standard deviation (SD). Assays used three samples per group (*n* = 3). **C** To assess metastasis suppression and the anti-tumor effects of first-line ovarian cancer therapy combined with a TGase 2 inhibitor, we performed tests using an orthotopic model using OVCAR‑8/Luc cells. Pharmacological inhibition of TGase 2 works synergistically with conventional chemotherapies and prolongs survival in an ovarian cancer mouse model. Mice (*n* ≥ 8 per group) received phosphate-buffered saline (PBS), streptonigrin (STN; 0.01 mg/kg), DDP (1 mg/kg), PTX (1 mg/kg), STN + DDP, or STN + PTX. Kaplan–Meier survival curves are shown; median survival was calculated using Kaplan–Meier statistics. **D** Quantification of histological scores for GSK3β in ovarian cancer patient tissue using inForm™ image analysis software. Tumors were classified as stage I (*n* = 83), stage II (*n* = 24), stage III–IV (*n* = 37), or metastatic lesions (*n* = 36). Representative immunohistochemical (IHC) images are included (Supplementary Fig. 5) scale bar, 100 µm. **E** Comparison of TG2 and GSK3β expression patterns during tumor development and metastasis. The TMA analysis graphs are presented as mean ± standard error of the mean (SEM). Multiple comparisons among three or more groups were performed with one-way analysis of variance (ANOVA). Statistical significance was set at **p < 0.01, ***p < 0.001, and ****p < 0.0001. ns, not significant.

To evaluate whether pharmacologic blockade of epithelial-mesenchymal transition (EMT) through TGase 2 inhibition is therapeutically beneficial, we conducted two preclinical mouse studies. First, SKOV-3/Luc cells were injected into the tail vein of BALB/c nude mice, and streptonigrin (or vehicle) was administered orally, starting 2 days before cell inoculation. After 4 weeks of treatment, the mice were observed for an additional 4 weeks (Fig. 5C). Streptonigrin treatment reduced SKOV-3 metastatic lesions in a dose-dependent manner, with H&E staining revealing a 70–84% decrease in the number of metastatic nodules and tumor area compared to the control group. To determine whether the anti-metastatic effect of streptonigrin correlates with the increased GSK3β expression observed in *TGM2* KO tissues, immunohistochemical staining was performed using tumors from SKOV-3 xenografts after streptonigrin treatment. Compared with the control group, GSK3β expression levels in tumor tissues increased by approximately 30% in the 0.01 mpk streptonigrin group and by 55% in the 0.1 mpk group (Supplementary Fig. 4). Next, we investigated whether targeting the TGase 2 N-terminus with streptonigrin can suppress ovarian cancer growth in an intraperitoneal xenograft model (Fig. 5D). OVCAR-8/Luc cells were injected intraperitoneally into BALB/c-nude mice, followed by oral administration of streptonigrin at doses of 0.01 or 0.1 mg/kg. Over a 5-week treatment period, streptonigrin significantly inhibited tumor growth in a dose-dependent manner. After treatment ended, survival was monitored until day 65. Control mice had a mean survival time of 51.5 days. Mice treated with 0.01 mg/kg streptonigrin survived for 58.5 days (7 days longer than controls), whereas none of the mice in the 0.1 mg/kg group died by day 65 (Fig. 5E). Because chemotherapy often fails by inducing EMT and drug resistance, we investigated whether TGase 2 inhibition could counteract these effects. Exposure of ovarian cancer cells to cisplatin (DDP) or paclitaxel (PTX) increased TGase 2 protein expression and concomitantly reduced GSK3β levels (Fig. 6A). Consistent with an EMT phenotype, both drugs increased cell migration by 10–20% in OVCAR‑3 cells and by 25–30% in SKOV‑3 cells (Fig. 6B). Based on these observations, we tested whether streptonigrin works synergistically with standard drugs in vivo (Fig. 6C). OVCAR‑8/Luc xenografts were established via intraperitoneal injection, and mice received streptonigrin (0.01 mg/kg, oral, daily) along with either cisplatin (1 mg/kg, i.p., weekly) or paclitaxel (1 or 10 mg/kg, i.p., weekly). The median survival in controls was 46.5 days. Monotherapy with paclitaxel (1 mg/kg) increased survival to 55.5 days, and cisplatin to 51 days. In contrast, combination therapy with streptonigrin and paclitaxel extended median survival to 73.5 days (18 days longer than with paclitaxel alone), with some mice living up to 80 days. Similarly, mice receiving streptonigrin along with cisplatin survived up to 80 days, with an average of 70.5 days (19.5 days longer than with cisplatin alone) (Fig. 6C). In summary, *TGM2* knockdown (*TGM2* k/d) (Fig. 4) or TGase 2 inhibition using streptonigrin (Fig. 5) increased the median survival of ovarian cancer. Notably, combining TGase 2 inhibition with cisplatin or paclitaxel significantly increased median survival by over 2.7 weeks compared to chemotherapy alone (Fig. 6C). Furthermore, to investigate changes in GSK3β expression during ovarian cancer progression, immunohistochemical analysis was performed using high-density ovarian cancer tissue arrays (Fig. 6D and E, Supplementary Fig. 5). GSK3β expression was higher in tumor tissues compared to normal tissues; however, statistical significance was only observed in stage I samples. Interestingly, GSK3β levels gradually decreased from stage II to stage III and were lowest in metastatic lesions. This expression pattern was compared with the TGase 2 staining results shown in Fig. 1. While TGase 2 expression significantly increased during tumor progression and metastasis, GSK3β expression showed the opposite trend, being progressively downregulated. These findings suggest that inhibiting TGase 2 presents a promising therapeutic strategy to improve the efficacy of existing chemotherapeutic treatments against ovarian cancer. These data emphasize TGase 2 inhibition as a promising strategy to improve the effectiveness of current chemotherapies for ovarian cancer.

## Discussion

Gase 2 supports two main features of ovarian cancer: increased invasiveness and multidrug resistance. These phenotypes are closely interconnected and are mainly controlled by specific signaling pathways, especially the WNT/β-catenin pathway [30, 31]. Under physiological conditions, active GSK3β suppresses cell invasion by promoting ubiquitin-mediated degradation of β-catenin. However, sustained β‑catenin signaling in tumors amplified both growth and dissemination [32, 33]. In ovarian cancer, abnormal activation of receptor tyrosine kinases (RTKs), including epidermal growth factor receptor (EGFR), fibroblast growth factor receptor (FGFR), insulin-like growth factor receptor (IGFR), and vascular endothelial growth factor receptor (VGFR), triggers the PI3K/AKT pathway. This activation then inactivates GSK3β, increases β-catenin activity, and upregulates invasion-related genes such as N-cadherin, fibronectin, and Snail [33, 34]. Consequently, PI3K/AKT inhibitors are undergoing clinical evaluation to control β‑catenin–dependent invasion [32, 34].

Our data reveal that blocking the TGase 2–GSK3β interaction is a viable strategy to suppress metastasis. Previously, we demonstrated in renal-cell carcinoma (RCC) that TGase 2 N-terminal region binds to p53 and that streptonigrin occupies the same region, displacing p53 and causing apoptosis [29, 35–37]. TGase 2 regulates intracellular p53 by facilitating its transport from the cytosol to autophagosomes via its N-terminal domain [38]. Notably, TGase 2 also stabilizes extracellular proteins, such as fibronectin, through its β-sandwich domain, which interacts with the 45-kDa gelatin-binding region of fibronectin [39, 40]. Deletion mapping localized this interaction surface to residues 81-140 of the first β-sandwich repeat [41]. Complementary site-directed mutagenesis and surface plasmon resonance analyses identified critical residues (K30, R116, and H134) essential for this interaction [11]. Medicinal chemistry efforts have therefore focused on small molecules that block TGase 2–fibronectin binding rather than the enzyme’s catalytic transamidase activity [13]. Intriguingly, the fibronectin‑binding surface overlaps both the p53‑binding stretch (1–139 aa) and the GSK3β‑binding motif (118–134 aa) [37, 42]. These findings suggest that small molecules targeting the N-terminal region of TGase 2 could disrupt TGase 2-GSK3β interaction, thereby inhibiting EMT through suppression of β-catenin activity [13].

Streptomycin has been reported to bind to the 95–116 amino acid residues of TGase 2 [28]. By preventing the formation of the TGase 2-p53 complex in renal cancer cells, streptomycin enhances p53 stability and triggers cell death. This occurs because 96% of renal cancer tumors retain wild-type p53 [28, 43]. However, restoring p53 levels through *TGM2* downregulation is less relevant in ovarian cancer. This is because p53 mutations occur at a high frequency of 100% in high-grade serous ovarian cancer [44]. Instead, blocking the action of TGase 2-GSK3β is vital in ovarian cancer. Our study found that TGase 2 directly and intracellularly regulates GSK3β to maintain β-catenin activity, which promotes cell invasion and increases cancer cell survival. Although basal TGase 2 expression varies from negligible to very high across cancer cell lines, it can be strongly induced by NF-κB in response to chemotherapeutics (Fig. 6A). Previous studies have shown that various modalities, including chemotherapy, radiotherapy, and hormonal therapy, activated NF-κB, which subsequently induced *TGM2* transcription [45, 46]. It has been reported that cytotoxic cancer drugs such as paclitaxel, doxorubicin, vinblastine, daunomycin, and vincristine induce NF-κB activation [47], which is closely associated with anti-cancer drug resistance [48]. Tyrosine kinase inhibitors also induce oxidative stress [49], resulting in NF-κB activation [50]. The increased TGase 2 expression from NF-κB activation can perpetuate NF-κB activation through I-κBα depletion via crosslinking, which was proposed as a term of “the TGase 2 Ouroboros loop” [17, 19, 20, 51]. Therefore, anti-cancer or inflammatory stress can upregulate TGase 2 even in cells that were previously negative for TGase 2. Unlike temporary kinase cascades, TGase 2 maintains sustained NF-κB activation, which enhances invasiveness and survival. Paradoxically, standard chemotherapy may promote EMT by inducing TGase 2. These findings indicate that blocking TGase 2 should be considered obligatory, not optional, in ovarian cancer treatment.

In this study, streptonigrin restored β-catenin suppression by stabilizing GSK3β through inhibiting the TGase 2-GSK3β interaction. This increased the median survival of mice with ovarian cancer treated with streptonigrin. These results are consistent with the survival outcomes observed in xenograft models using *TGM2* knockout cell lines compared to wild-type controls. Here, we demonstrated that the ligands for the N-terminus region of TGase 2 can disrupt TGase 2-GSK3β complexes and improve outcomes for ovarian cancer.

## Materials and methods

### Cell lines and cell cultures

Ovarian Cancer cell lines, including OVCAR3 (HTB-161), SKOV-3 (HTB-77), and HEK-293 (CRL-1573) were obtained from the American Type Culture Collection (ATCC, Manassas, VA, USA). Ovarian cancer cell lines OVCAR-4, OVCAR-5, and OVCAR-8 were obtained from the National Cancer Institute (Material Transfer Agreement number: 2702-09). All cell lines were authenticated by Short Tandem Repeat (STR) profiling within six months before experimentation (KCLB, Seoul, Republic of Korea) and tested negative for mycoplasma, Sendai virus, and mouse hepatitis virus by PCR (Lonza, LT07-318). No cell lines listed in the ICLAC register of misidentified lines were used. HEK-293 cells were cultured at 37 °C in complete Dulbecco’s Modified Eagle Medium (DMEM) (Cytiva, UT, USA) supplemented with 10% fetal bovine serum (Cytiva, UT, USA) under a humidified atmosphere of 5% CO2. All ovarian cancer cells were cultured at 37 °C in complete Roswell Park Memorial Institute (RPMI) 1640 medium (Cytiva, UT, USA) supplemented with 10% fetal bovine serum (Cytiva, UT, USA) under a humidified atmosphere of 5% CO_2_. SKOV-3 cells overexpressing firefly luciferase (SKOV-3/luc) were generated as previously described [52]. For OVCAR-8 cells overexpressing firefly luciferase (OVCAR-8/luc), cells were transfected with the pGL4.51 (luc2/CMV/Neo) firefly luciferase reporter plasmid (Promega, Madison, WI, USA) using Lipofectamine 3000 (Thermo Fisher Scientific, Waltham, MA, USA). Stably transfected clones were selected by culturing in 100 μg/mL of G418, and luciferase expression was confirmed using the Dual-Luciferase Reporter Assay System (Promega). Luminescence was measured with a Victor Luminometer (PerkinElmer, Waltham, MA, USA). *TGM2* knockout OVCAR-8/luc cells were generated using the CRISPR Cas9 lentiviral system. The scrambled sgRNA CRISPR/Cas9 All-in-One Lentivector (#K010) and *TGM2* sgRNA CRISPR/Cas9 All-in-One Lentivector set (#K2366205) for human cells were purchased from Applied Biological Materials Inc. (Richmond, BC, Canada). Cell lines were established following the manufacturer’s protocols.

### Antibodies and reagents

Antibodies Anti-TGase 2 (#PA5-23219, #MA5-12736) and anti-HA-tag (#71-5500) were purchased from Thermo Scientific; anti-β-actin (#sc-47778), anti-N-cadherin (#sc-7939), anti-Fibronectin (#sc-8422), anti-Vimentin (#sc-6260) and anti-HA-tag (#sc-7392) were from Santa Cruz Biotechnology (Dallas, TX, USA); anti-GSK3β (#12456), anti-p-GSK3β (Ser9) (#9323), anti-non-p (Active) β-catenin (Ser33/37/Thr41) (#8814) and anti-Snail (#3879) were from Cell Signaling Technology (Beverly, MA, USA); anti-FLAG-tag antibody (#F1804, #F7425) was from Sigma-Aldrich (St. Louis, MO, USA). Reagents: Streptonigrin (#S1014) and cis-Diammineplatinum (II) dichloride (cisplatin; #P4394) were purchased from Sigma-Aldrich; paclitaxel (#S1150) was from Selleck Chemicals LLC (Houston, TX, USA); GK921 was synthesized by Professor Young-Dae Gong (Dongguk University, Seoul, Republic of Korea) [53]; INTERFERin® (#409-50) and jetPEI® (#101-40 N) transfection reagents were from PolyPlus-transfection (Illkirch-Graffenstaden, France); small interfering RNA (siRNA) duplex targeting human TGase 2 and scrampled siRNA were from GenePharma (Shanghai, China). All antibodies used in this study were validated either by the manufacturers or internally using siRNA knockdown to confirm specificity. Research Resource Identifiers (RRIDs) for key antibodies are as follows: anti-TGase 2 (PA5-23219, RRID:AB_2545257), anti-GSK3β (12456, RRID:AB_2636978), and anti-β-actin (sc-47778, RRID:AB_2714189).

### Immunoblotting

Cells were lysed in Radioimmunoprecipitation assay (RIPA) buffer, and protein concentrations were determined using the bicinchoninic acid (BCA) protein assay kit (Pierce, Rockford, IL, USA). Equal amounts of total protein (10-40 μg per lane) were separated via SDS-polyacrylamide gel electrophoresis (SDS-PAGE) and transferred to polyvinylidene fluoride (PVDF) membranes. All immunoblottings were performed with the same amount of protein per sample set after protein quantification. The optimal immunoblottings exposure time varies depending on the target and cell line, therefore, immunoblottings were performed on separate gels for each set. The membranes were blocked for 1 h at room temperature with 5% bovine serum albumin (BSA) in Tris-buffered saline (TBS) containing 0.1% (v/v) Tween 20 (TBST). Following the blocking step, membranes were incubated for 1 h 30 min at room temperature with primary antibodies diluted 1:1000 in 5% BSA. After three washes with TBST, the membranes were incubated for 1 h at room temperature with horseradish peroxidase (HRP)-conjugated secondary antibody. Membranes were then washed five times with TBST, and protein signals were detected using Westsave™ chemiluminescent substrate (AbFrontier, Seoul, Korea). Gels images were captured with a FUSION-Solo.4.WL imaging system (Vilber Lourmat, Marne-la-Vallée, France).

### Co-immunoprecipitation

To evaluate whether TGase 2 binds to GSK3β, OVCAR-8 cells were transfected with *TGM2* siRNA or scramble siRNA. To identify the region of TGase 2 required for GSK3β binding, HEK-293 cells were co-transfected with HA-tagged wild-type *TGM2* or one of several *TGM2* deletion mutants (Δ2-139, Δ147-460, Δ472-583, Δ592-673) together with FLAG-tagged *GSK3β*. To determine which specific GSK3β residues are necessary for binding to TGase 2, HEK-293 cells were co-transfected with HA-tagged wild-type *GSK3β* or one of the *GSK3β* mutants (Y216F, V267G/E268R, F291L), along with FLAG-tagged *TGM2*. Finally, to assess whether streptonigrin inhibits the interaction of TGase 2 with GSK3β, HEK-293 cells co-transfected with HA-tagged *GSK3β* and FLAG-tagged *TGM2* were treated with various concentrations of streptonigrin for 1 h prior to harvesting. All transfected cells were cultured for 48 h and then lysed using immunoprecipitation buffer (50 mM Tris-HCl, 150 mM NaCl, 1 mM EDTA, 0.5% Triton X-100, pH 7.4) supplemented with a protease inhibitor cocktail. The lysates were incubated with anti-HA or anti-FLAG antibodies (1 μg/mL) at 4°C overnight in an immunoprecipitation buffer. Next, 10 μL of protein A/G UltraLink Resin beads (50:50 resin-to-buffer slurry; Pierce, #35133) were added to each sample and incubated at room temperature for 2 h. Immunoprecipitates were collected by centrifuging at 3000 rpm for 3 min, followed by five washes in 500 μl of immunoprecipitation buffer with gentle tapping between washes. The final immunoprecipitates were then analyzed by Western blot.

### Immunofluorescence analysis

OVCAR-8 cells were cultured on sterile coverslips and treated with streptonigrin (500 nM; Sigma-Aldrich, Cat. No. S3389) for 1 h. After treatment, cells were fixed with cold methanol for 30 min at −20 °C and washed with phosphate-buffered saline (PBS). Fixed cells were incubated overnight at 4 °C with primary antibodies against TGase 2 (Cat. No. 68006-1-Ig; 1:800 dilution, Proteintech) and GSK3β (Cat. No. #12456; 1:500 dilution, Cell Signaling Technology). After washing, cells were incubated for 1 h at room temperature with Alexa Fluor 488–conjugated anti-mouse IgG secondary antibody (Invitrogen, Cat. No. A-21202; 1:1000 dilution) and Alexa Fluor 594–conjugated anti-rabbit IgG secondary antibody (Invitrogen, Cat. No. A-11012; 1:1,000 dilution). Nuclei were counterstained with DAPI (Hoechst 33342, Invitrogen, Cat. No. H21492; 1:2,000 dilution) for 5 min. Confocal imaging was performed using an Axiovert 200 M laser scanning confocal microscope (Carl Zeiss, Germany). Co-localization of TGase 2 and GSK3β was analyzed using ZEN software (Carl Zeiss).

### Cell Migration and Invasion assay

Cell migration and invasion were assessed using 6.5 mm Transwell chambers fitted with 8.0 μm pore filters (Costar, Cambridge, MA, USA). For migration assays, various concentrations of streptonigrin were added to the lower chamber of each well. SKOV-3 cells (1 × 10^5^ cells per well) or OVCAR-8 (3 × 10^5^ cells per well) cells were seeded into the upper Transwell inserts. After incubation at 37 °C for 8 h (SKOV-3) or 48 h (OVCAR-8), the inserts were removed and stained using a Diff-Quick staining kit (Sysmex, Kobe, Japan). Migrated cells were visualized by light microscopy. For invasion assays, Transwell inserts were precoated with Matrigel (1 mg/mL; BD BioSciences, San Jose, CA, USA) to mimic the extracellular matrix. OVCAR-8 cells (2 × 10⁵ cells per well) and SKOV3 cells (0.5 × 10⁵ cells per well) were seeded in the Matrigel-coated inserts and incubated for 48 h at 37 °C. The inserts were stained with a Diff-Quick staining kit, and invasive cells were visualized under a light microscope.

### Intraperitoneal xenograft mouse model of ovarian cancer

To assess early tumor growth and intraperitoneal dissemination of ovarian cancer, we employed an orthotopic model using the OVCAR-8 cell line. All animal studies were reviewed and approved by the Institutional Animal Care and Use Committee (IACUC, NCC-18-452 and NCC-19-486) of the National Cancer Center, Republic of Korea. The National Cancer Center Research Institute (NCCRI) is an AAALAC International-accredited facility that adheres to the guidelines of the Institute of Laboratory Animal Resources (ILAR). To evaluate the effect of TGM2 on ovarian cancer progression, 7-week-old female BALB/c-nude mice (*n* = 12 per group; Orient Bio, Seongnam, Korea) were intraperitoneally injected with 1 × 10⁷ OVCAR-8/Luc or OVCAR-8/Luc TGM2 knockout (KO) cells in 200 μL of PBS.

To assess the efficacy of streptonigrin, additional groups of mice inoculated with OVCAR-8/Luc cells were treated orally with PBS, 0.01, or 0.1 mg/kg streptonigrin, administered five days per week starting two days before tumor implantation, with animals randomly assigned to experimental groups. For combination treatment experiments, mice were administered either PBS or 0.01 mg/kg streptonigrin orally (five days per week). Beginning four days post-inoculation, they were given 1 mg/kg cisplatin or paclitaxel intravenously once a week. Tumor progression was monitored noninvasively by bioluminescence imaging (IVIS) rather than by caliper measurements because the model involved intraperitoneal dissemination. The maximum permissible tumor burden, defined as a total luminescence intensity not exceeding 1 × 10⁹ photons/sec, was established in accordance with IACUC and institutional ethical guidelines. Blinding could not be implemented because of equipment and staffing constraints and the need to process multiple groups simultaneously. To mitigate potential bias, all groups were maintained under identical housing and imaging conditions, and data were analyzed according to a pre-specified analysis pipeline with pre-defined exclusion criteria. Humane endpoints including >20% body weight loss, abdominal distension, or impaired mobility were predefined, and no animals exceeded these limits or required early euthanasia. Mice were intraperitoneally injected with 75 mg/kg luciferin, and images were captured using an In Vivo Imaging System (IVIS; Caliper Life Science, Waltham, MA, USA). Bioluminescence signals were quantified using Living Image software with standardized square regions of interest (ROIs) applied consistently across all measurements. The survival of animals was monitored for up to 80 days following tumor implantation to evaluate differences between the control and experimental groups. Statistical significance between groups was assessed using the log-rank test.

### Tail vein injection and lung metastasis assay

To evaluate lung metastasis, a tail vein injection protocol was employed using luciferase-expressing human ovarian cancer cells. Ovarian cancer cells were cultured under standard conditions and harvested at approximately 80% confluence. Cells were washed twice with sterile PBS, resuspended in PBS at a concentration of 1 × 10⁶ cells per 100 µL, and kept on ice until injection. Each BALB/c-nude mouse (6–8 weeks old, Female, *n* = 4) received an intravenous injection of 100 µL of the cell suspension via the lateral tail vein using a 26-gauge insulin syringe. The sample size (*n* = 4) was chosen based on previous reports using IVIS-based metastatic models, where 3–5 animals per group were sufficient to achieve consistent luminescent detection of pulmonary metastases. Animal studies involving streptonigrin were performed with random allocation to groups. Because this study aimed to confirm metastatic colonization rather than to perform statistical comparison, four animals per group were deemed adequate, in compliance with the 3Rs principle of minimizing animal use. Metastasized tumors were collected and analyzed by immunohistochemistry.

### Automated immunohistochemistry

Immunohistochemistry (IHC) was performed on formalin-fixed paraffin-embedded (FFPE) lung tissues from mice and ovarian cancer patient tissue microarray (TMA) sections. An ovarian cancer tissue array (OV208a) was purchased from Tissue Array (Derwood, MD, US). Briefly, the sections were deparaffinized in xylene, dehydrated in ethanol, and rehydrated with water. Antigen retrieval was performed by immersing the slides in antigen retrieval buffer (10 mM sodium citrate, 0.05% Tween 20, pH 6.0) at 95 °C for 5 min. Endogenous peroxidases were blocked with 0.03% hydrogen peroxide, and nonspecific binding was blocked with 2% bovine serum albumin (BSA) in Tris-buffered saline with 0.1% Triton X-100 (TBST, pH 7.6) for 30 min. The sections were incubated with the primary antibody for 1.5 h at room temperature. After washing, the sections were incubated with the 3,3-diaminobenzidine chromogen (DAB) system to develop the stain for 5–20 min. The sections were counterstained with Mayer’s hematoxylin for 30–60 s. Finally, the sections were dehydrated through 95% ethanol, followed by 100% ethanol, cleared in xylene, and mounted. The primary antibodies used in this experiment, their sources, and dilution ratios were as follows: GSK3β (27C10) Rabbit mAb (Cat No. 9315S, 1:400) from Cell Signaling Technology (Danvers, MA, US). TGase 2 (Cat No. PA5-23219, 1:500 from Thermo Fisher Scientific (Waltham, MA, USA). Tissue microarray cores and animal tumor tissues were included when tissue integrity was preserved (no folding, dropout, or extensive necrosis) and staining conditions were uniform. Slides with optical artifacts (wrinkles, bubbles), analyzable tumor area <1 mm², or automated analysis QC score <80/100 after re-analysis were excluded.

### Image acquisition and analysis

The stained human ovarian cancer TMA was scanned in high-resolution mode using the Vectra Polaris multispectral imaging system (Akoya Biosciences, Marlborough, MA, USA) and Motic Easy Scan digital slide scanner (Motic, Kowloon Bay, Hong Kong). Protein expression was analyzed based on the pathological evaluation of tissue morphology and staining patterns in ovarian cancer tissues. The inForm Image Analysis Software (Akoya Biosciences, Marlborough, MA, USA) was used to quantitatively assess the IHC results. H-scores were calculated based on the percentage of positively stained cells and staining intensity. The significance of TGase 2 or GSK3β expression across different groups was analyzed using one-way ANOVA in GraphPad Prism version 10.3.1.

### Prediction of GSK3-TGase 2 interaction

To predict the structural model for the GSK3β-TGase 2 interaction, the ClusPro server (https://cluspro.org) was used. Crystal structures of GSK3β (PDB code: 4NM5) and TGase 2 (PDB code: 2Q3Z) served as templates [42, 54]. ClusPro identifies centers of highly populated clusters of low-energy docked conformations based on each energy parameter set, thus generating the most probable complex models.

### Correlation analysis

Correlation analyses were performed using the GEPIA2 web server (http://gepia2.cancer-pku.cn), where pairwise gene expression correlations were calculated using Spearman’s rank correlation. Gene sets for each category were obtained from PMID: 23251436. Correlation coefficients (R) and p-values were obtained directly from GEPIA2, and the results were visualized as scatter plots using log2-transformed TPM values.

### Statistical analysis

For all in vitro assays, at least three independent biological replicates were performed, with each measurement repeated in triplicate (technical replicates). Data points shown represent biological replicates unless otherwise specified. Sample size (n) for each experimental group was determined based on prior power calculations using G*Power 3.1 software (α = 0.05, power = 0.8, expected effect size = 0.8). For xenograft experiments, group size (*n* = 12) was selected to ensure adequate power while minimizing animal use according to the 3Rs principle. Where blinding could not be implemented due to operational constraints, bias mitigation included uniform husbandry/imaging conditions and analysis under a pre-specified pipeline with predefined exclusion criteria. All statistical analyses were performed using the GraphPad Prism software <version 10.6.0.> (GraphPad Software Inc., San Diego, CA, USA). Survival rates in xenograft models were evaluated using Kaplan–Meier plots, with significance assessed by the log-rank test. Comparisons between two groups were made using a two-tailed Student’s *t*-test, assuming equal variances unless otherwise stated, whereas one-way or two-way analysis of variance (ANOVA) was employed for multiple comparisons. For tail-vein lung-metastasis assays (*n* = 4 per group), group comparisons were performed using two-tailed exact Mann–Whitney tests (or Kruskal–Wallis with Dunn’s post-hoc correction for ≥3 groups), given the small sample size and non-normality concerns; effect sizes (Cliff’s delta) with 95% bootstrap confidence intervals were reported, and plots display individual animal values with median and interquartile range (IQR). Statistical significance was set as *p < 0.05, **p < 0.01, or ***p < 0.001, ****p < 0.0001, ns, not significant.

### Ethical approval

The informed consent is not relevant to this study because an ovarian cancer tissue array (OV208a) was purchased from Tissue Array (Derwood, MD, US). Since we did not collect human specimens, IRB approval is also irrelevant to this study. We confirm that all methods were performed in accordance with the relevant guidelines and regulations. All animal studies were reviewed and approved by the Institutional Animal Care and Use Committee (IACUC, NCC-18-452 and NCC-19-486) of the National Cancer Center, Republic of Korea. The National Cancer Center Research Institute (NCCRI) is an AAALAC International-accredited facility that adheres to the guidelines of the Institute of Laboratory Animal Resources (ILAR).

## Supplementary information

Supplementary Figures

## Data Availability

There is no data beyond what is presented in the manuscript. Please contact the corresponding author directly to request information regarding the experimental results in the manuscript, and we will provide it.
