# Supplementary material for: Transglutaminase 2 exacerbates ovarian cancer survival by directly inactivating GSK3β
Source: Cell Death Dis. 2026 Feb 2;17(1):199. doi: 10.1038/s41419-026-08447-0 (PMC12876850; doi:10.1038/s41419-026-08447-0)
Supplement: Supplementary file 1 — Supplementary Figures [file 41419_2026_8447_MOESM1_ESM.pdf]

A

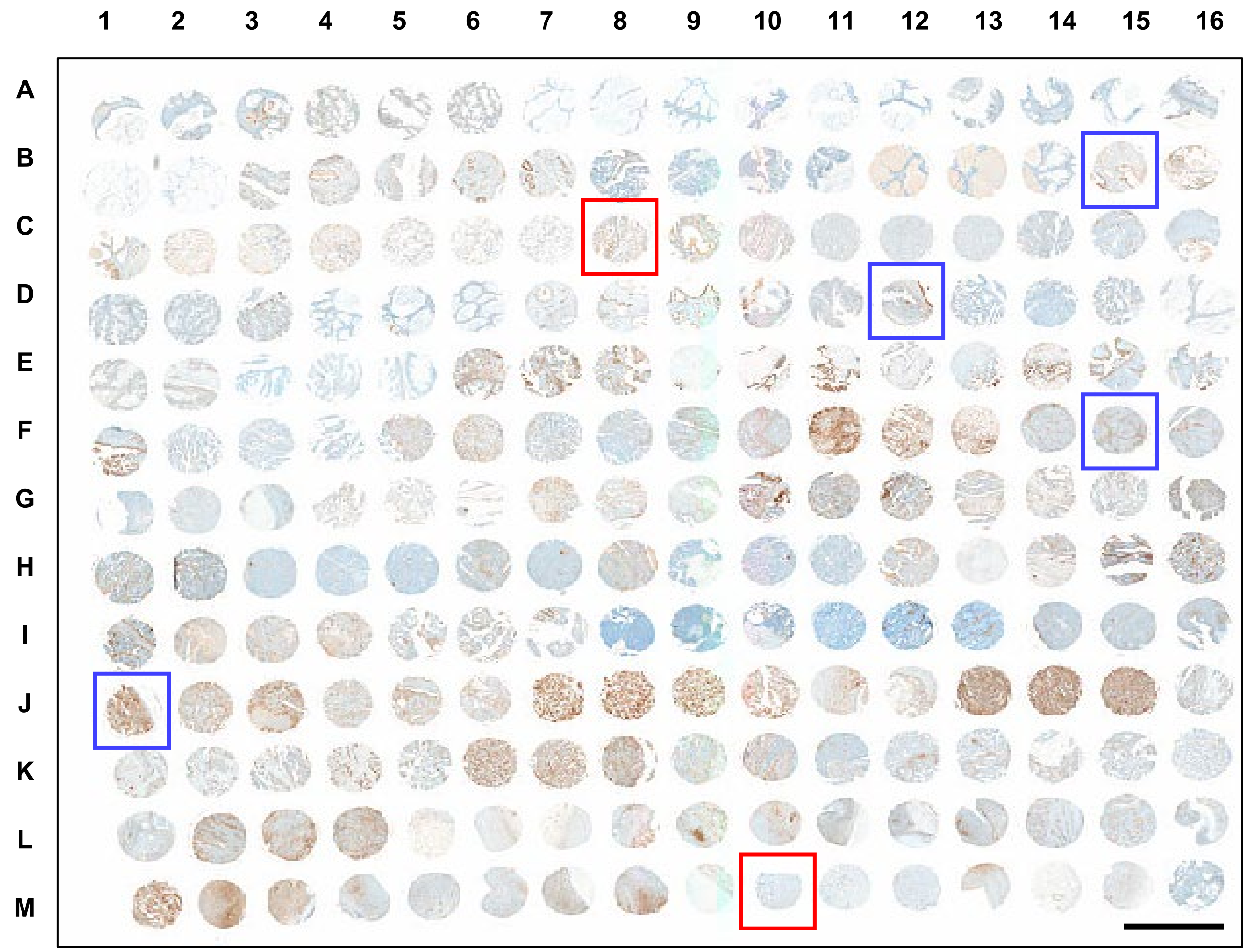

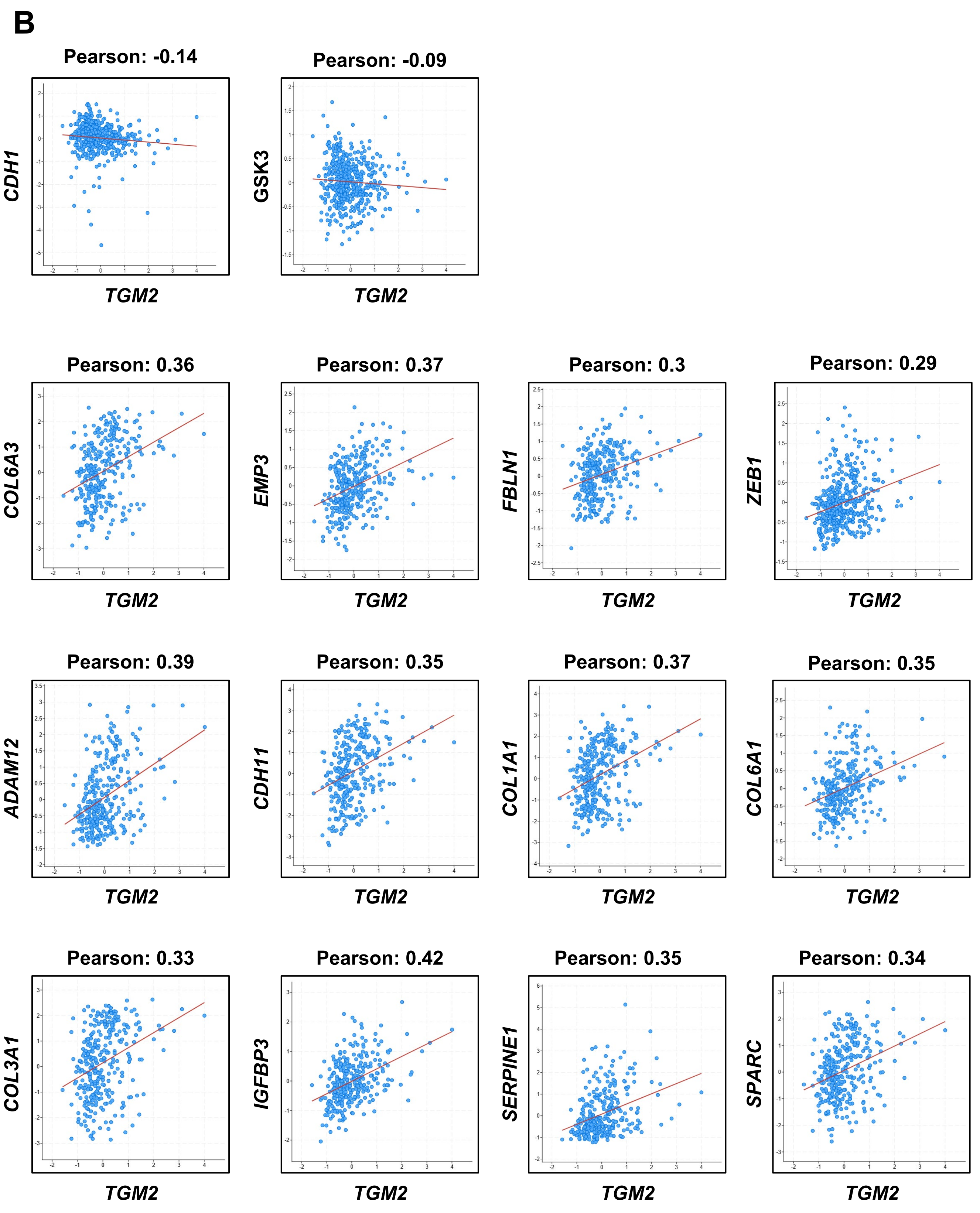
B
Pearson: -0.14
CDH1
TGM2
Pearson: -0.09
GSK3
TGM2
Pearson: 0.36
COL6A3
TGM2
Pearson: 0.37
EMP3
TGM2
Pearson: 0.3
FBLN1
TGM2
Pearson: 0.29
ZEB1
TGM2
Pearson: 0.39
ADAM12
TGM2
Pearson: 0.35
CDH11
TGM2
Pearson: 0.37
COL1A1
TGM2
Pearson: 0.35
COL6A1
TGM2
Pearson: 0.33
COL3A1
TGM2
Pearson: 0.42
IGFBP3
TGM2
Pearson: 0.35
SERPINE1
TGM2
Pearson: 0.34
SPARC
TGM2

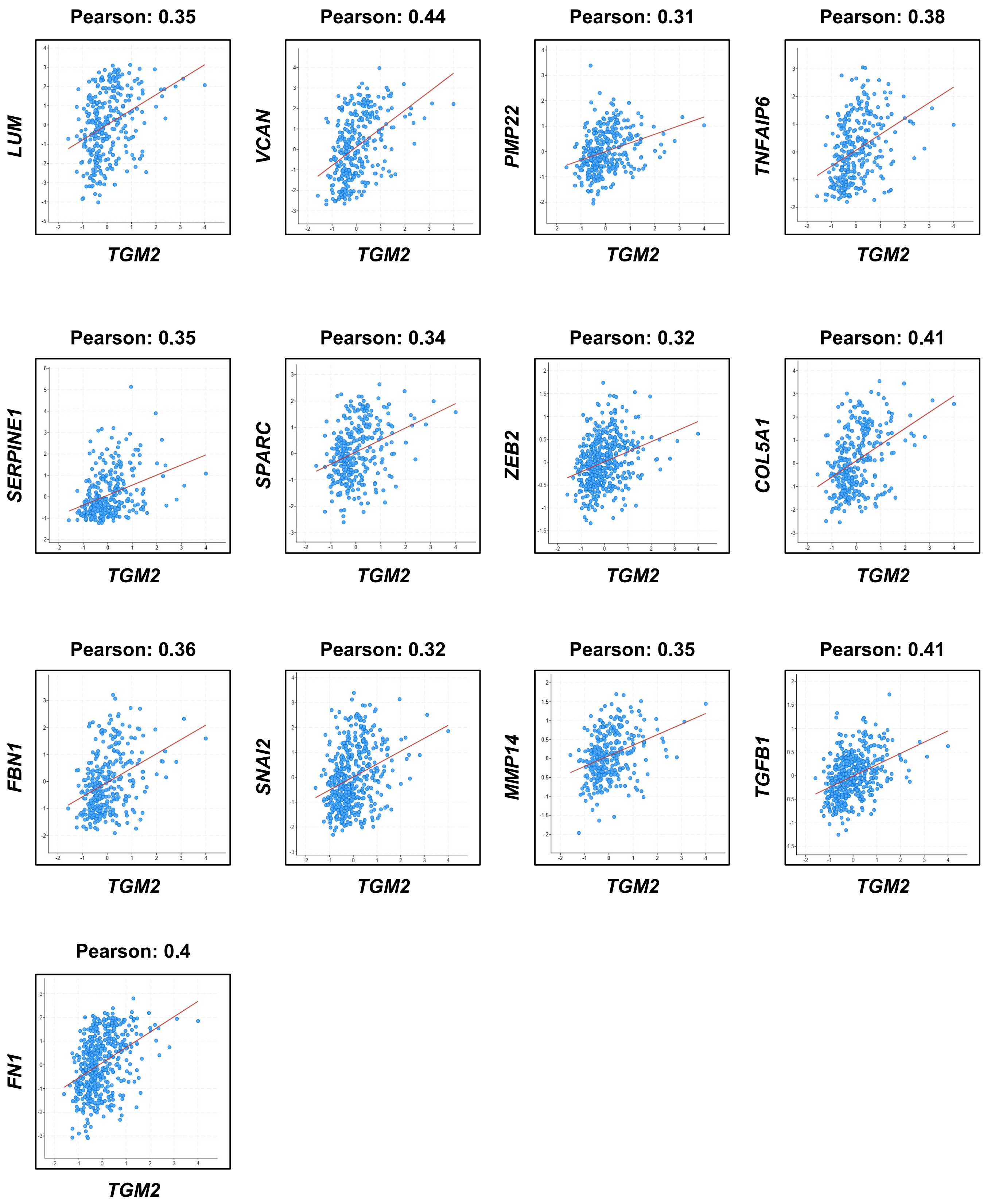

Supplementary Figure 1. TGase 2 expression in OVC TMA. A Ovarian cancer patient tissue microarray (TMA) containing 69 cases/207 cores within normal tissue samples, with triplicate cores per case (OV208a), was purchased from Tissue Array (Derwood, MD, US). IHC images of TMA (OV208a) stained with anti-TG2 (PA5-23219). Red boxes highlight the images shown in Figure 1*a*. Blue boxes highlight the images shown in Figure 1b. (Scale bar = 2 mm). **B** Scatter plot showing the correlation between *TGM2* mRNA abundance and the expression of key epithelial–mesenchymal transition (EMT) genes.

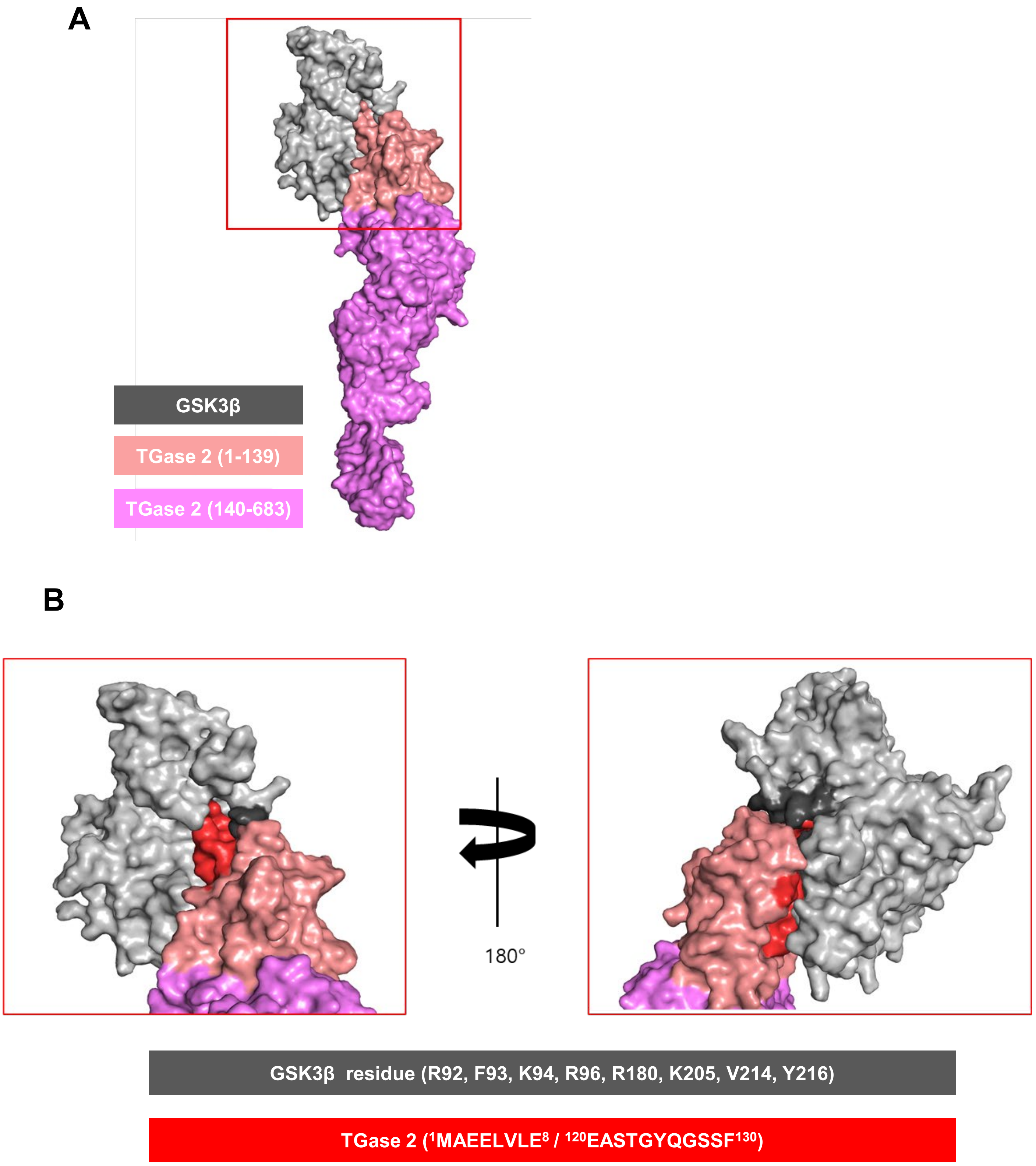

Supplementary Figure 2. Direct binding of TGase 2 and GSK3β. **A** Overall structure of docking result. The GSK3β (PDB code 4NM5) is drawn in gray, and the TGase 2 (PDB code 2Q3Z) is drawn in apricot (residues 1-139) and pink (residues 140-683), respectively. **B** Binding site of the GSK3 β (black-gray) and the TGase 2(red) were predicted by the ClusPro server.

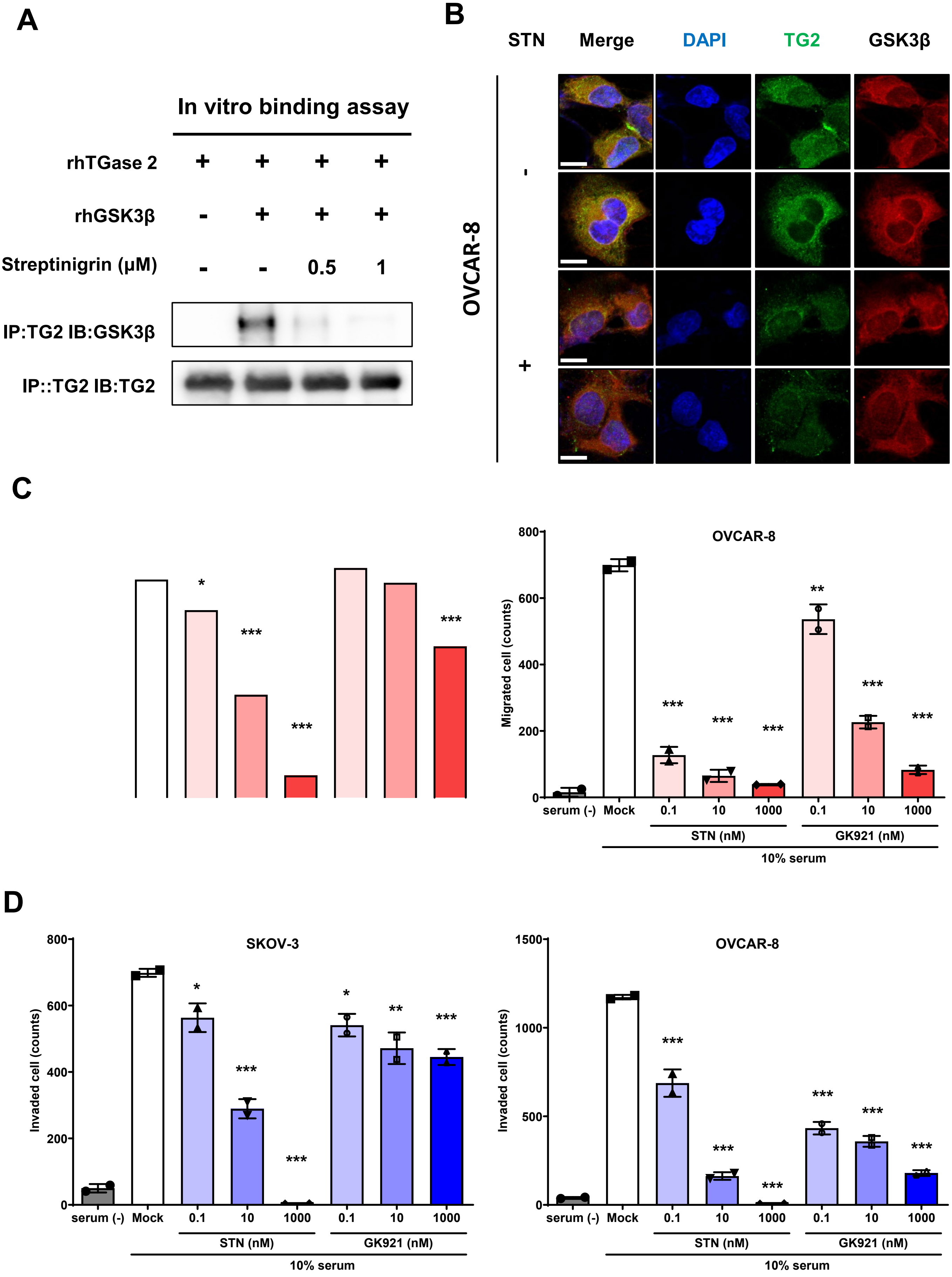
A
In vitro binding assay
rhTGase 2 + + + +
rhGSK3β - + + +
Streptinigrin (μM) - - 0.5 1
IP:TG2 IB:GSK3β
IP::TG2 IB:TG2
B
STN
Merge
DAPI
TG2
GSK3β
OVCAR-8
-
+
C
*
***
***
***
OVCAR-8
Migrated cell (counts)
800
600
400
200
0
**
***
***
***
***
***
serum (-)
Mock
0.1
10
1000
0.1
10
1000
STN (nM)
GK921 (nM)
10% serum
D
SKOV-3
Invaded cell (counts)
800
600
400
200
0
*
***
***
*
**
***
serum (-)
Mock
0.1
10
1000
0.1
10
1000
STN (nM)
GK921 (nM)
10% serum
OVCAR-8
Invaded cell (counts)
1500
1000
500
0
***
***
***
***
***
***
serum (-)
Mock
0.1
10
1000
0.1
10
1000
STN (nM)
GK921 (nM)
10% serum

Supplementary Figure 3. TGase 2 inhibitors suppress migration and invasion of ovarian cancer cells. A The interaction between TGase 2 and GSK3β was examined using recombinant proteins. Each protein (500 ng) was used for the assay. For inhibition, TGase 2 was pre-incubated with streptonigrin, a TGase 2 inhibitor, on ice for 30 min prior to the addition of GSK3β. After a final incubation at room temperature for 30 min, immunoprecipitation (IP) was performed. **B** streptonigirn reduces intracellular co-localization of TGase 2 and GSK3β. OVCAR-8 cells were exposed to 500 nM STN for 24 h and analyzed by confocal microscopy. Scale bar, 20 μm. **C** Effects of TGase 2 inhibitors on the migration of SKOV-3 and OVCAR-8 ovarian cancer cells. For the migration assay, the upper and lower chambers of Transwell inserts were coated with fibronectin. After 1 h, cells that had migrated to the underside of the filter were fixed, stained, and quantified as described in Materials and Methods. **D** Effects of TGase 2 inhibitors on the invasion of SKOV-3 and OVCAR-8 cells. For the invasion assay, Transwell chambers were coated with Matrigel. SKOV-3 and OVCAR-8 cells were treated with streptonigrin[1] (0.1, 10, and 1000 nM) or GK921[2] (0.1, 10, and 1000 nM). After 24 h, invasive cells on the membrane underside were fixed, stained, and counted as described in Materials and Methods. *$p<0.05$, **$p < 0.01$, and ***$p < 0.001$.

1. Lee SH, Lee WK, Kim N, et al. Renal Cell Carcinoma Is Abrogated by p53 Stabilization through Transglutaminase 2 Inhibition. Cancers (Basel) 2018; 10(11).
2. Kim N, Kang JH, Lee WK, et al. Allosteric inhibition site of transglutaminase 2 is unveiled in the N terminus. Amino Acids 2018; 50(11): 1583-94.

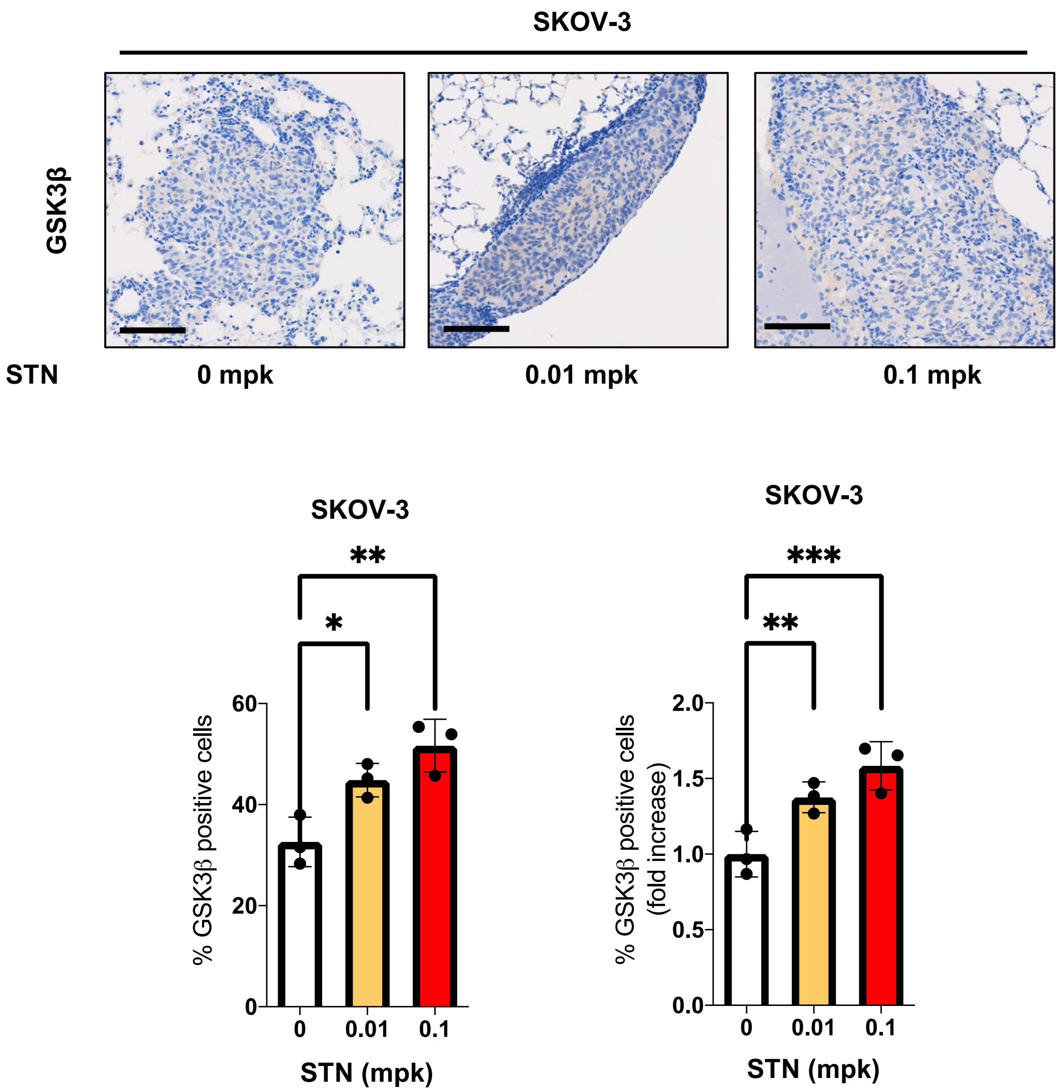

Supplementary Figure 4. TGase 2 inhibitor treatment induces GSK3β level in lung metastasis tissue. GSK3β expression in SKOV-3 tumor was anlayzed after streptonigrin treatment. Quantitation of GSK3β was conducted using the Vectra Polaris™ Automated Quantitative Pathology Imaging System and inForm software (Akoya Biosciences, Waltham, MA). Scale bar, 100 μm. The graphs are displayed as mean ± standard deviation (SD). Experiments were performed with three samples per group (n = 3). Comparisons between two groups were performed using the Student's t-test. Statistical significance was defined as *$p < 0.05$ or **$p < 0.01$ or ***$p < 0.001$, and ns indicates not significant.

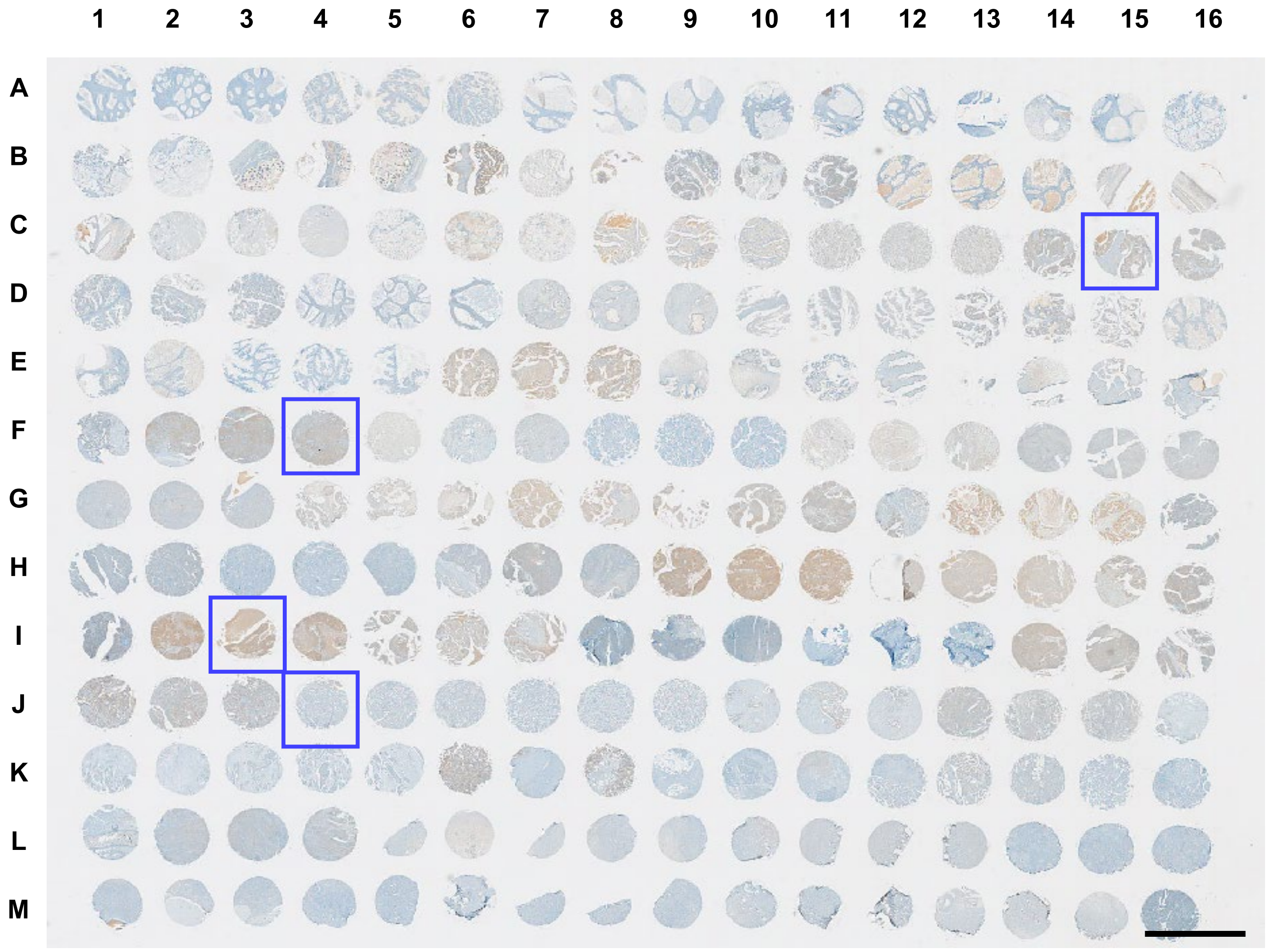

Supplementary Figure 5. GSK3β expression in OVC TMA. Ovarian cancer patient tissue microarray (TMA) containing 69 cases/207 cores within normal tissue samples, with triplicate cores per case (OV208a), was purchased from Tissue Array (Derwood, MD, US). IHC images of TMA (OV208a) stained with anti- GSK3β (#9315, cell signaling). Blue boxes highlight the images shown in Figure 6D (Scale bar = 2 mm).
